# Supplementary material for: Dissemination of antibiotic resistance genes from antibiotic producers to pathogens
Source: Nat Commun. 2017 Jun 7;8:15784. doi: 10.1038/ncomms15784 (PMC5467266; doi:10.1038/ncomms15784)
Supplement: Supplementary Information — Supplementary Figures and Supplementary Table [file ncomms15784-s1.pdf]

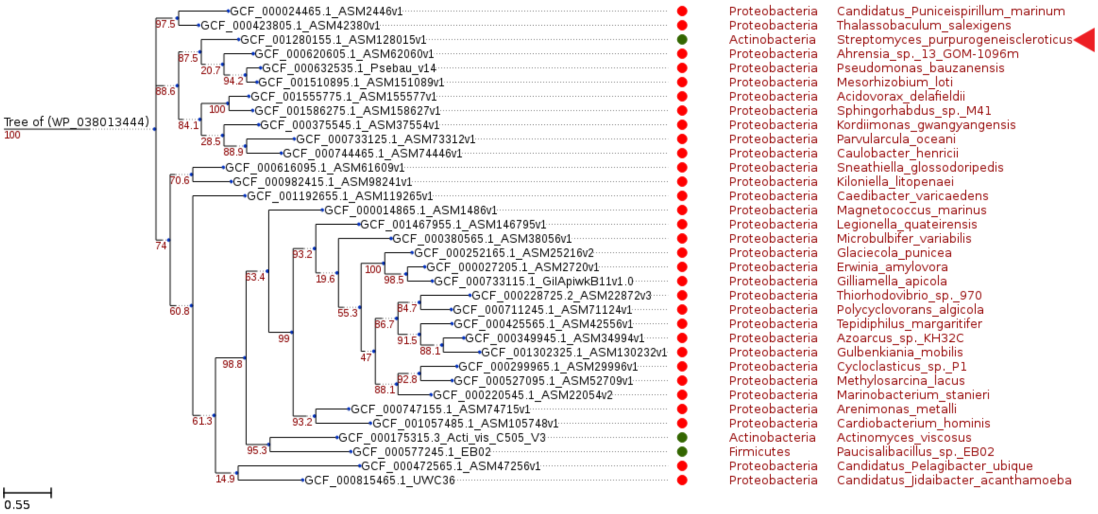
Tree of (WP_038013444)
100
GCF_000024465.1_ASM2446v1 Proteobacteria Candidatus_Puniceispirillum_marinum
GCF_000423805.1_ASM42380v1 Proteobacteria Thalassobaculum_salexigens
GCF_001280155.1_ASM128015v1 Actinobacteria Streptomyces_purpurogeneiscleroticus
GCF_000620605.1_ASM62060v1 Proteobacteria Ahrensia_sp._13_GOM-1096m
GCF_000632535.1_Psebau_v14 Proteobacteria Pseudomonas_bauzanensis
GCF_001510895.1_ASM151089v1 Proteobacteria Mesorhizobium_loti
GCF_001555775.1_ASM155577v1 Proteobacteria Acidovorax_delafieldii
GCF_001586275.1_ASM158627v1 Proteobacteria Sphingorhabdus_sp._M41
GCF_000375545.1_ASM37554v1 Proteobacteria Kordiimonas_gwangyangensis
GCF_000733125.1_ASM73312v1 Proteobacteria Parvularcula_oceani
GCF_000744465.1_ASM74446v1 Proteobacteria Caulobacter_henricii
GCF_000616095.1_ASM61609v1 Proteobacteria Sneathiella_glossodoripedis
GCF_000982415.1_ASM98241v1 Proteobacteria Kiloniella_litopenaei
GCF_001192655.1_ASM119265v1 Proteobacteria Caedibacter_varicaedens
GCF_000014865.1_ASM1486v1 Proteobacteria Magnetococcus_marinus
GCF_001467955.1_ASM146795v1 Proteobacteria Legionella_quateirensis
GCF_000380565.1_ASM38056v1 Proteobacteria Microbulbifer_variabilis
GCF_000252165.1_ASM25216v2 Proteobacteria Glaciecola_punicea
GCF_000027205.1_ASM2720v1 Proteobacteria Erwinia_amylovora
GCF_000733115.1_GilApiwkB11v1.0 Proteobacteria Gilliamella_apicola
GCF_000228725.2_ASM22872v3 Proteobacteria Thiorhodovibrio_sp._970
GCF_000711245.1_ASM71124v1 Proteobacteria Polycyclovorans_algicola
GCF_000425565.1_ASM42556v1 Proteobacteria Tepidiphilus_margaritifer
GCF_000349945.1_ASM34994v1 Proteobacteria Azoarcus_sp._KH32C
GCF_001302325.1_ASM130232v1 Proteobacteria Gulbenkiania_mobilis
GCF_000299965.1_ASM29996v1 Proteobacteria Cycloclasticus_sp._P1
GCF_000527095.1_ASM52709v1 Proteobacteria Methylosarcina_lacus
GCF_000220545.1_ASM22054v2 Proteobacteria Marinobacterium_stanieri
GCF_000747155.1_ASM74715v1 Proteobacteria Arenimonas_metalli
GCF_001057485.1_ASM105748v1 Proteobacteria Cardiobacterium_hominis
GCF_000175315.3_Acti_vis_C505_V3 Actinobacteria Actinomyces_viscosus
GCF_000577245.1_EB02 Firmicutes Paucisalibacillus_sp._EB02
GCF_000472565.1_ASM47256v1 Proteobacteria Candidatus_Pelagibacter_ubique
GCF_000815465.1_UWC36 Proteobacteria Candidatus_Jidaibacter_acanthamoeba
0.55

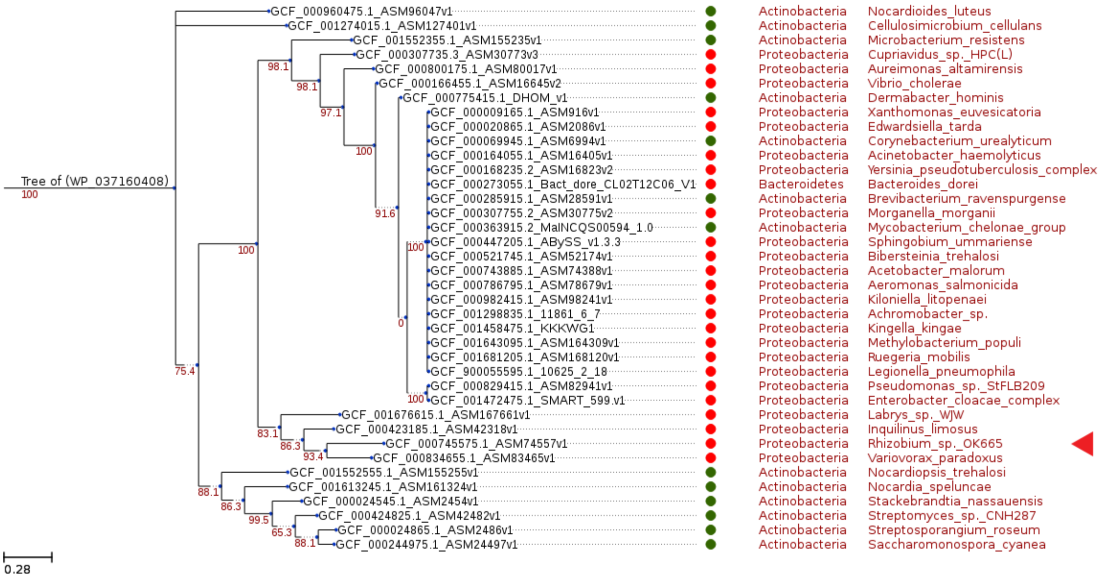
Tree of (WP_037160408)
100
GCF_000960475.1_ASM96047v1 Actinobacteria Nocardioides_luteus
GCF_001274015.1_ASM127401v1 Actinobacteria Cellulosimicrobium_cellulans
GCF_001552355.1_ASM155235v1 Actinobacteria Microbacterium_resistens
GCF_000307735.3_ASM30773v3 Proteobacteria Cupriavidus_sp._HPC(L)
GCF_000800175.1_ASM80017v1 Proteobacteria Aureimonas_altamirensis
GCF_000166455.1_ASM16645v2 Proteobacteria Vibrio_cholerae
GCF_000775415.1_DHOM_v1 Actinobacteria Dermabacter_hominis
GCF_000009165.1_ASM916v1 Proteobacteria Xanthomonas_euvesicatoria
GCF_000020865.1_ASM2086v1 Proteobacteria Edwardsiella_tarda
GCF_000069945.1_ASM6994v1 Actinobacteria Corynebacterium_urealyticum
GCF_000164055.1_ASM16405v1 Proteobacteria Acinetobacter_haemolyticus
GCF_000168235.2_ASM16823v2 Proteobacteria Yersinia_pseudotuberculosis_complex
GCF_000273055.1_Bact_dore_CL02T12C06_V1 Bacteroidetes Bacteroides_dorei
GCF_000285915.1_ASM28591v1 Actinobacteria Brevibacterium_ravenspurgense
GCF_000307755.2_ASM30775v2 Proteobacteria Morganella_morganii
GCF_000363915.2_MalNCQS00594_1.0 Actinobacteria Mycobacterium_chelonae_group
GCF_000447205.1_ABySS_v1.3.3 Proteobacteria Sphingobium_ummariense
GCF_000521745.1_ASM52174v1 Proteobacteria Bibersteinia_trehalosi
GCF_000743885.1_ASM74388v1 Proteobacteria Acetobacter_malorum
GCF_000786795.1_ASM78679v1 Proteobacteria Aeromonas_salmonicida
GCF_000982415.1_ASM98241v1 Proteobacteria Kiloniella_litopenaei
GCF_001298835.1_11861_6_7 Proteobacteria Achromobacter_sp.
GCF_001458475.1_KKKWG1 Proteobacteria Kingella_kingae
GCF_001643095.1_ASM164309v1 Proteobacteria Methylobacterium_populi
GCF_001681205.1_ASM168120v1 Proteobacteria Ruegeria_mobilis
GCF_900055595.1_10625_2_18 Proteobacteria Legionella_pneumophila
GCF_000829415.1_ASM82941v1 Proteobacteria Pseudomonas_sp._StFLB209
GCF_001472475.1_SMART_599.v1 Proteobacteria Enterobacter_cloacae_complex
GCF_001676615.1_ASM167661v1 Proteobacteria Labrys_sp._WJW
GCF_000423185.1_ASM42318v1 Proteobacteria Inquilinus_limosus
GCF_000745575.1_ASM74557v1 Proteobacteria Rhizobium_sp._OK665
GCF_000834655.1_ASM83465v1 Proteobacteria Variovorax_paradoxus
GCF_001552555.1_ASM155255v1 Actinobacteria Nocardiopsis_trehalosi
GCF_001613245.1_ASM161324v1 Actinobacteria Nocardia_speluncae
GCF_000024545.1_ASM2454v1 Actinobacteria Stackebrandtia_nassauensis
GCF_000424825.1_ASM42482v1 Actinobacteria Streptomyces_sp._CNH287
GCF_000024865.1_ASM2486v1 Actinobacteria Streptosporangium_roseum
GCF_000244975.1_ASM24497v1 Actinobacteria Saccharomonospora_cyanea
0.28

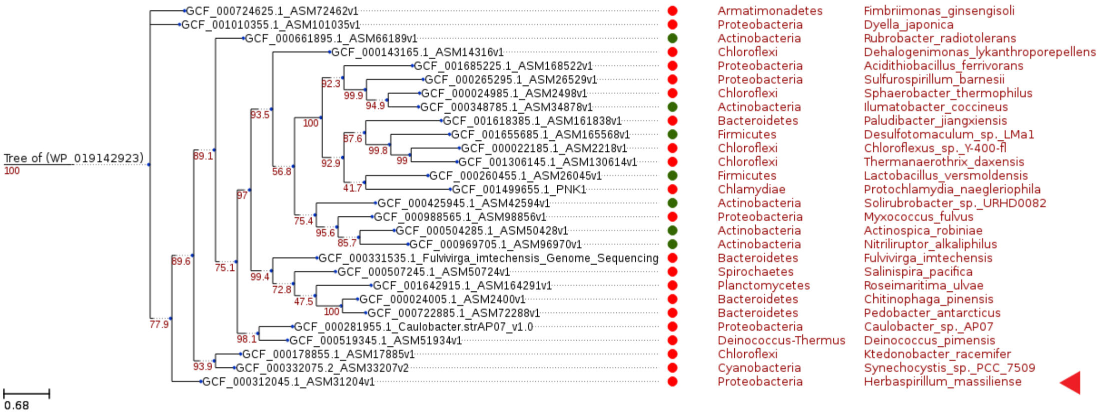
Tree of (WP_019142923)
100
GCF_000724625.1_ASM72462v1 Armatimonadetes Fimbriimonas_ginsengisoli
GCF_001010355.1_ASM101035v1 Proteobacteria Dyella_japonica
GCF_000661895.1_ASM66189v1 Actinobacteria Rubrobacter_radiotolerans
GCF_000143165.1_ASM14316v1 Chloroflexi Dehalogenimonas_lykanthroporepellens
GCF_001685225.1_ASM168522v1 Proteobacteria Acidithiobacillus_ferrivorans
GCF_000265295.1_ASM26529v1 Proteobacteria Sulfurospirillum_barnesii
GCF_000024985.1_ASM2498v1 Chloroflexi Sphaerobacter_thermophilus
GCF_000348785.1_ASM34878v1 Actinobacteria Ilumatobacter_coccineus
GCF_001618385.1_ASM161838v1 Bacteroidetes Paludibacter_jiangxiensis
GCF_001655685.1_ASM165568v1 Firmicutes Desulfotomaculum_sp._LMa1
GCF_000022185.1_ASM2218v1 Chloroflexi Chloroflexus_sp._Y-400-fl
GCF_001306145.1_ASM130614v1 Chloroflexi Thermanaerothrix_daxensis
GCF_000260455.1_ASM26045v1 Firmicutes Lactobacillus_versmoldensis
GCF_001499655.1_PNK1 Chlamydiae Protochlamydia_naegleriophila
GCF_000425945.1_ASM42594v1 Actinobacteria Solirubrobacter_sp._URHD0082
GCF_000988565.1_ASM98856v1 Proteobacteria Myxococcus_fulvus
GCF_000504285.1_ASM50428v1 Actinobacteria Actinospica_robiniae
GCF_000969705.1_ASM96970v1 Actinobacteria Nitriliruptor_alkaliphilus
GCF_000331535.1_Fulvivirga_imtechensis_Genome_Sequencing Bacteroidetes Fulvivirga_imtechensis
GCF_000507245.1_ASM50724v1 Spirochaetes Salinispira_pacifica
GCF_001642915.1_ASM164291v1 Planctomycetes Roseimaritima_ulvae
GCF_000024005.1_ASM2400v1 Bacteroidetes Chitinophaga_pinensis
GCF_000722885.1_ASM72288v1 Bacteroidetes Pedobacter_antarcticus
GCF_000281955.1_Caulobacter.strAP07_v1.0 Proteobacteria Caulobacter_sp._AP07
GCF_000519345.1_ASM51934v1 Deinococcus-Thermus Deinococcus_pimensis
GCF_000178855.1_ASM17885v1 Chloroflexi Ktedonobacter_racemifer
GCF_000332075.2_ASM33207v2 Cyanobacteria Synechocystis_sp._PCC_7509
GCF_000312045.1_ASM31204v1 Proteobacteria Herbaspirillum_massiliense
0.68

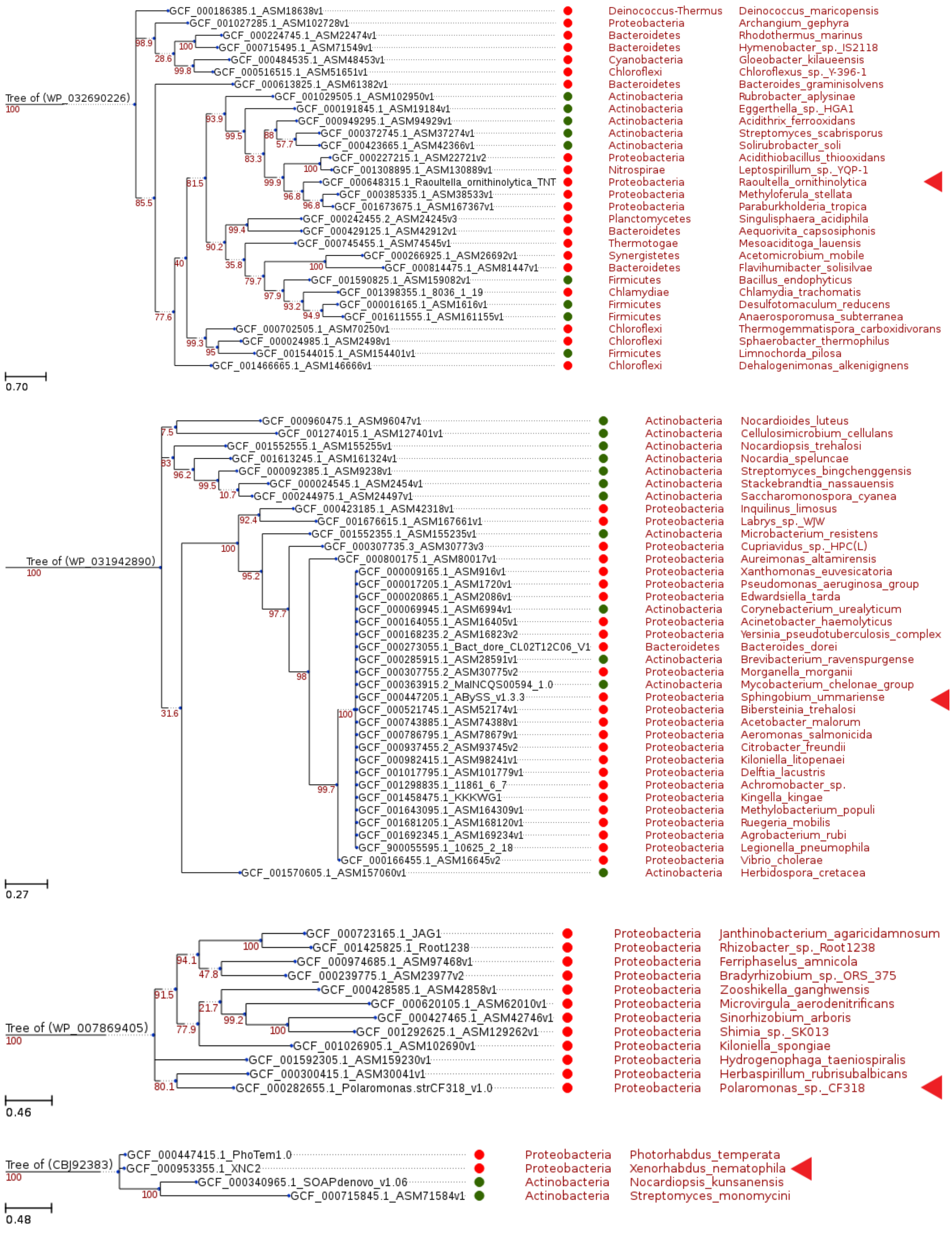

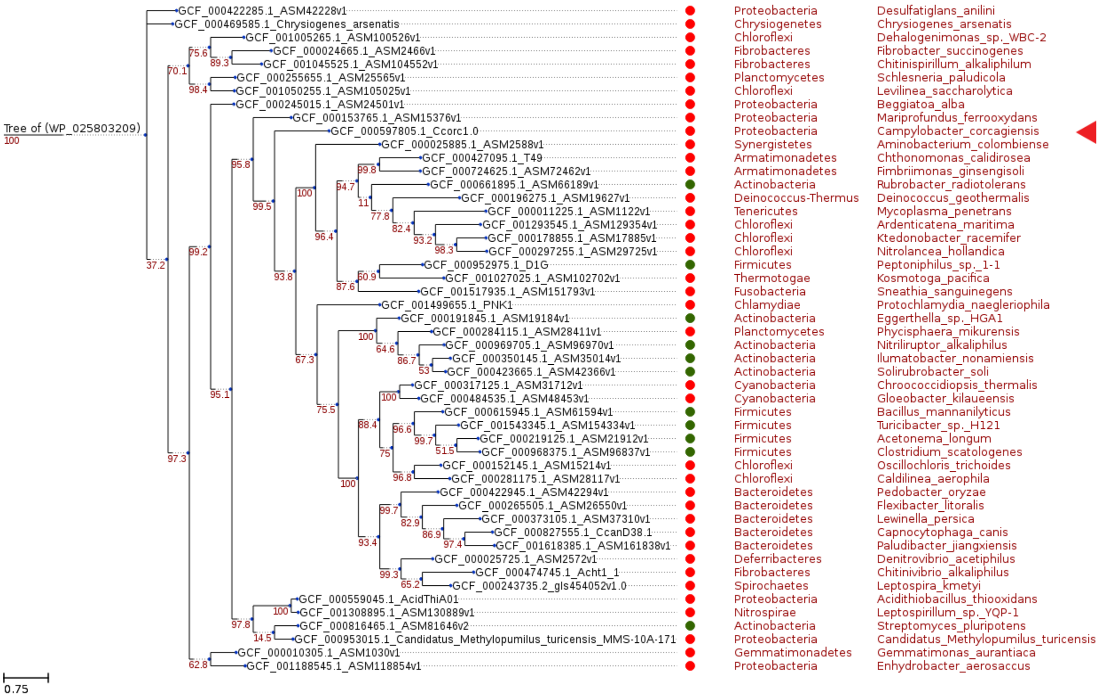
Tree of (WP_025803209)
100
0.75
GCF_000422285.1_ASM42228v1 Proteobacteria Desulfatiglans_anilini
GCF_000469585.1_Chrysiogenes_arsenatis Chrysiogenetes Chrysiogenes_arsenatis
GCF_001005265.1_ASM100526v1 Chloroflexi Dehalogenimonas_sp._WBC-2
GCF_000024665.1_ASM2466v1 Fibrobacteres Fibrobacter_succinogenes
GCF_001045525.1_ASM104552v1 Fibrobacteres Chitinispirillum_alkaliphilum
GCF_000255655.1_ASM25565v1 Planctomycetes Schlesneria_paludicola
GCF_001050255.1_ASM105025v1 Chloroflexi Levilinea_saccharolytica
GCF_000245015.1_ASM24501v1 Proteobacteria Beggiatoa_alba
GCF_000153765.1_ASM15376v1 Proteobacteria Mariprofundus_ferrooxydans
GCF_000597805.1_Ccorc1.0 Proteobacteria Campylobacter_corcagiensis
GCF_000025885.1_ASM2588v1 Synergistetes Aminobacterium_colombiense
GCF_000427095.1_T49 Armatimonadetes Chthonomonas_calidirosea
GCF_000724625.1_ASM72462v1 Armatimonadetes Fimbriimonas_ginsengisoli
GCF_000661895.1_ASM66189v1 Actinobacteria Rubrobacter_radiotolerans
GCF_000196275.1_ASM19627v1 Deinococcus-Thermus Deinococcus_geothermalis
GCF_000011225.1_ASM1122v1 Tenericutes Mycoplasma_penetrans
GCF_001293545.1_ASM129354v1 Chloroflexi Ardenticatena_maritima
GCF_000178855.1_ASM17885v1 Chloroflexi Ktedonobacter_racemifer
GCF_000297255.1_ASM29725v1 Chloroflexi Nitrolancea_hollandica
GCF_000952975.1_D1G Firmicutes Peptoniphilus_sp._1-1
GCF_001027025.1_ASM102702v1 Thermotogae Kosmotoga_pacifica
GCF_001517935.1_ASM151793v1 Fusobacteria Sneathia_sanguinegens
GCF_001499655.1_PNK1 Chlamydiae Protochlamydia_naegleriophila
GCF_000191845.1_ASM19184v1 Actinobacteria Eggerthella_sp._HGA1
GCF_000284115.1_ASM28411v1 Planctomycetes Phycisphaera_mikurensis
GCF_000969705.1_ASM96970v1 Actinobacteria Nitriliruptor_alkaliphilus
GCF_000350145.1_ASM35014v1 Actinobacteria Ilumatobacter_nonamiensis
GCF_000423665.1_ASM42366v1 Actinobacteria Solirubrobacter_soli
GCF_000317125.1_ASM31712v1 Cyanobacteria Chroococcidiopsis_thermalis
GCF_000484535.1_ASM48453v1 Cyanobacteria Gloeobacter_kilaueensis
GCF_000615945.1_ASM61594v1 Firmicutes Bacillus_mannanilyticus
GCF_001543345.1_ASM154334v1 Firmicutes Turicibacter_sp._H121
GCF_000219125.1_ASM21912v1 Firmicutes Acetonema_longum
GCF_000968375.1_ASM96837v1 Firmicutes Clostridium_scatologenes
GCF_000152145.1_ASM15214v1 Chloroflexi Oscillochloris_trichoides
GCF_000281175.1_ASM28117v1 Chloroflexi Caldilinea_aerophila
GCF_000422945.1_ASM42294v1 Bacteroidetes Pedobacter_oryzae
GCF_000265505.1_ASM26550v1 Bacteroidetes Flexibacter_litoralis
GCF_000373105.1_ASM37310v1 Bacteroidetes Lewinella_persica
GCF_000827555.1_CcanD38.1 Bacteroidetes Capnocytophaga_canis
GCF_001618385.1_ASM161838v1 Bacteroidetes Paludibacter_jiangxiensis
GCF_000025725.1_ASM2572v1 Deferribacteres Denitrovibrio_acetiphilus
GCF_000474745.1_Acht1_1 Fibrobacteres Chitinivibrio_alkaliphilus
GCF_000243735.2_gls454052v1.0 Spirochaetes Leptospira_kmetyi
GCF_000559045.1_AcidThiA01 Proteobacteria Acidithiobacillus_thiooxidans
GCF_001308895.1_ASM130889v1 Nitrospirae Leptospirillum_sp._YQP-1
GCF_000816465.1_ASM81646v2 Actinobacteria Streptomyces_pluripotens
GCF_000953015.1_Candidatus_Methylopumilus_turicensis_MMS-10A-171 Proteobacteria Candidatus_Methylopumilus_turicensis
GCF_000010305.1_ASM1030v1 Gemmatimonadetes Gemmatimonas_aurantiaca
GCF_001188545.1_ASM118854v1 Proteobacteria Enhydrobacter_aerosaccus

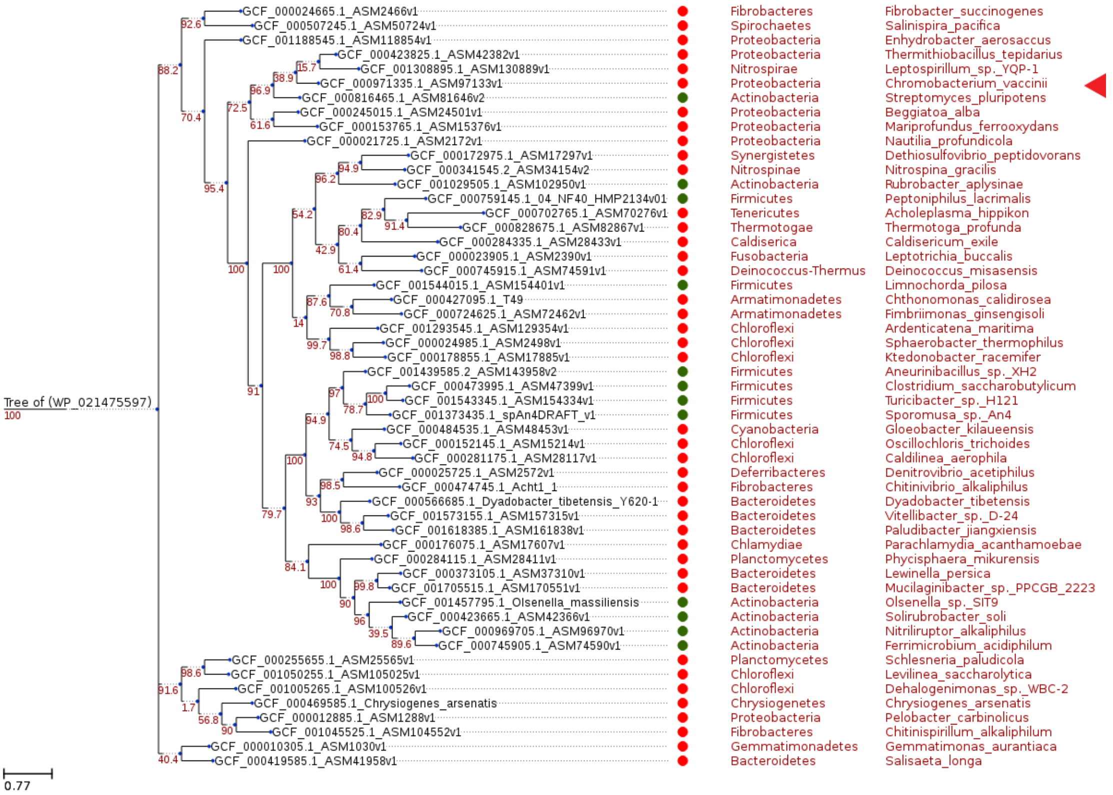
Tree of (WP_021475597)
100
0.77
GCF_000024665.1_ASM2466v1 Fibrobacteres Fibrobacter_succinogenes
GCF_000507245.1_ASM50724v1 Spirochaetes Salinispira_pacifica
GCF_001188545.1_ASM118854v1 Proteobacteria Enhydrobacter_aerosaccus
GCF_000423825.1_ASM42382v1 Proteobacteria Thermithiobacillus_tepidarius
GCF_001308895.1_ASM130889v1 Nitrospirae Leptospirillum_sp._YQP-1
GCF_000971335.1_ASM97133v1 Proteobacteria Chromobacterium_vaccinii
GCF_000816465.1_ASM81646v2 Actinobacteria Streptomyces_pluripotens
GCF_000245015.1_ASM24501v1 Proteobacteria Beggiatoa_alba
GCF_000153765.1_ASM15376v1 Proteobacteria Mariprofundus_ferrooxydans
GCF_000021725.1_ASM2172v1 Proteobacteria Nautilia_profundicola
GCF_000172975.1_ASM17297v1 Synergistetes Dethiosulfovibrio_peptidovorans
GCF_000341545.2_ASM34154v2 Nitrospinae Nitrospina_gracilis
GCF_001029505.1_ASM102950v1 Actinobacteria Rubrobacter_aplysinae
GCF_000759145.1_04_NF40_HMP2134v01 Firmicutes Peptoniphilus_lacrimalis
GCF_000702765.1_ASM70276v1 Tenericutes Acholeplasma_hippikon
GCF_000828675.1_ASM82867v1 Thermotogae Thermotoga_profunda
GCF_000284335.1_ASM28433v1 Caldiserica Caldisericum_exile
GCF_000023905.1_ASM2390v1 Fusobacteria Leptotrichia_buccalis
GCF_000745915.1_ASM74591v1 Deinococcus-Thermus Deinococcus_misasensis
GCF_001544015.1_ASM154401v1 Firmicutes Limnochorda_pilosa
GCF_000427095.1_T49 Armatimonadetes Chthonomonas_calidirosea
GCF_000724625.1_ASM72462v1 Armatimonadetes Fimbriimonas_ginsengisoli
GCF_001293545.1_ASM129354v1 Chloroflexi Ardenticatena_maritima
GCF_000024985.1_ASM2498v1 Chloroflexi Sphaerobacter_thermophilus
GCF_000178855.1_ASM17885v1 Chloroflexi Ktedonobacter_racemifer
GCF_001439585.2_ASM143958v2 Firmicutes Aneurinibacillus_sp._XH2
GCF_000473995.1_ASM47399v1 Firmicutes Clostridium_saccharobutylicum
GCF_001543345.1_ASM154334v1 Firmicutes Turicibacter_sp._H121
GCF_001373435.1_spAn4DRAFT_v1 Firmicutes Sporomusa_sp._An4
GCF_000484535.1_ASM48453v1 Cyanobacteria Gloeobacter_kilaueensis
GCF_000152145.1_ASM15214v1 Chloroflexi Oscillochloris_trichoides
GCF_000281175.1_ASM28117v1 Chloroflexi Caldilinea_aerophila
GCF_000025725.1_ASM2572v1 Deferribacteres Denitrovibrio_acetiphilus
GCF_000474745.1_Acht1_1 Fibrobacteres Chitinivibrio_alkaliphilus
GCF_000566685.1_Dyadobacter_tibetensis_Y620-1 Bacteroidetes Dyadobacter_tibetensis
GCF_001573155.1_ASM157315v1 Bacteroidetes Vitellibacter_sp._D-24
GCF_001618385.1_ASM161838v1 Bacteroidetes Paludibacter_jiangxiensis
GCF_000176075.1_ASM17607v1 Chlamydiae Parachlamydia_acanthamoebae
GCF_000284115.1_ASM28411v1 Planctomycetes Phycisphaera_mikurensis
GCF_000373105.1_ASM37310v1 Bacteroidetes Lewinella_persica
GCF_001705515.1_ASM170551v1 Bacteroidetes Mucilaginibacter_sp._PPCGB_2223
GCF_001457795.1_Olsenella_massiliensis Actinobacteria Olsenella_sp._SIT9
GCF_000423665.1_ASM42366v1 Actinobacteria Solirubrobacter_soli
GCF_000969705.1_ASM96970v1 Actinobacteria Nitriliruptor_alkaliphilus
GCF_000745905.1_ASM74590v1 Actinobacteria Ferrimicrobium_acidiphilum
GCF_000255655.1_ASM25565v1 Planctomycetes Schlesneria_paludicola
GCF_001050255.1_ASM105025v1 Chloroflexi Levilinea_saccharolytica
GCF_001005265.1_ASM100526v1 Chloroflexi Dehalogenimonas_sp._WBC-2
GCF_000469585.1_Chrysiogenes_arsenatis Chrysiogenetes Chrysiogenes_arsenatis
GCF_000012885.1_ASM1288v1 Proteobacteria Pelobacter_carbinolicus
GCF_001045525.1_ASM104552v1 Fibrobacteres Chitinispirillum_alkaliphilum
GCF_000010305.1_ASM1030v1 Gemmatimonadetes Gemmatimonas_aurantiaca
GCF_000419585.1_ASM41958v1 Bacteroidetes Salisaeta_longa

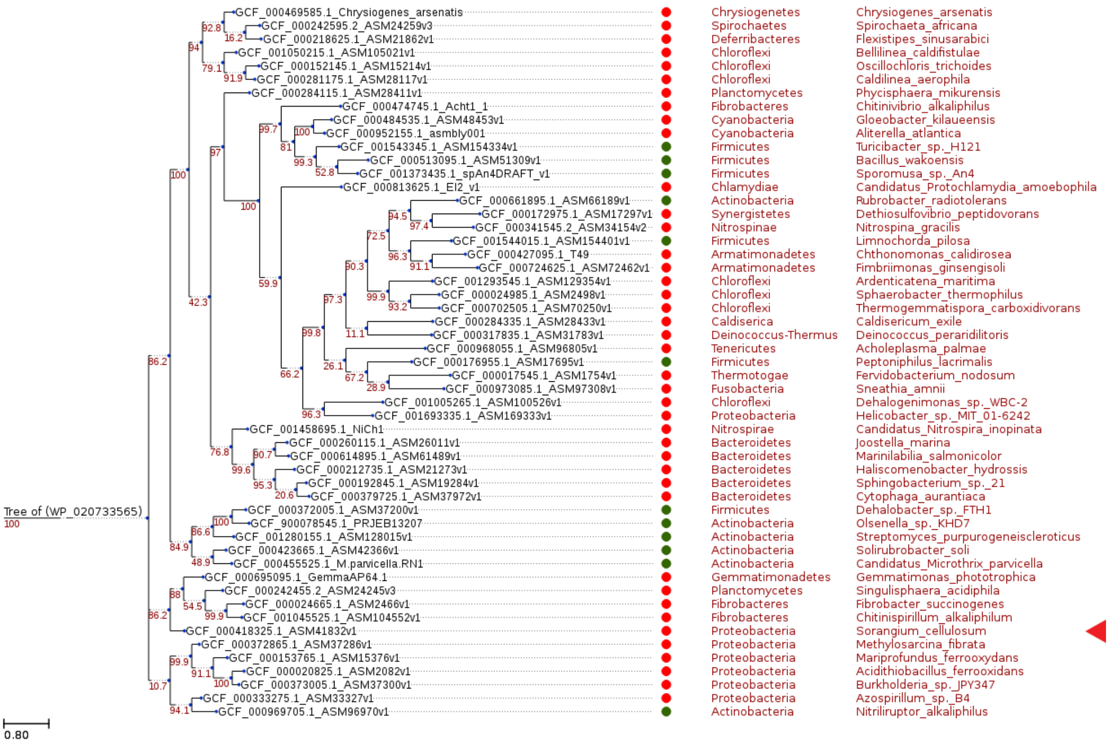

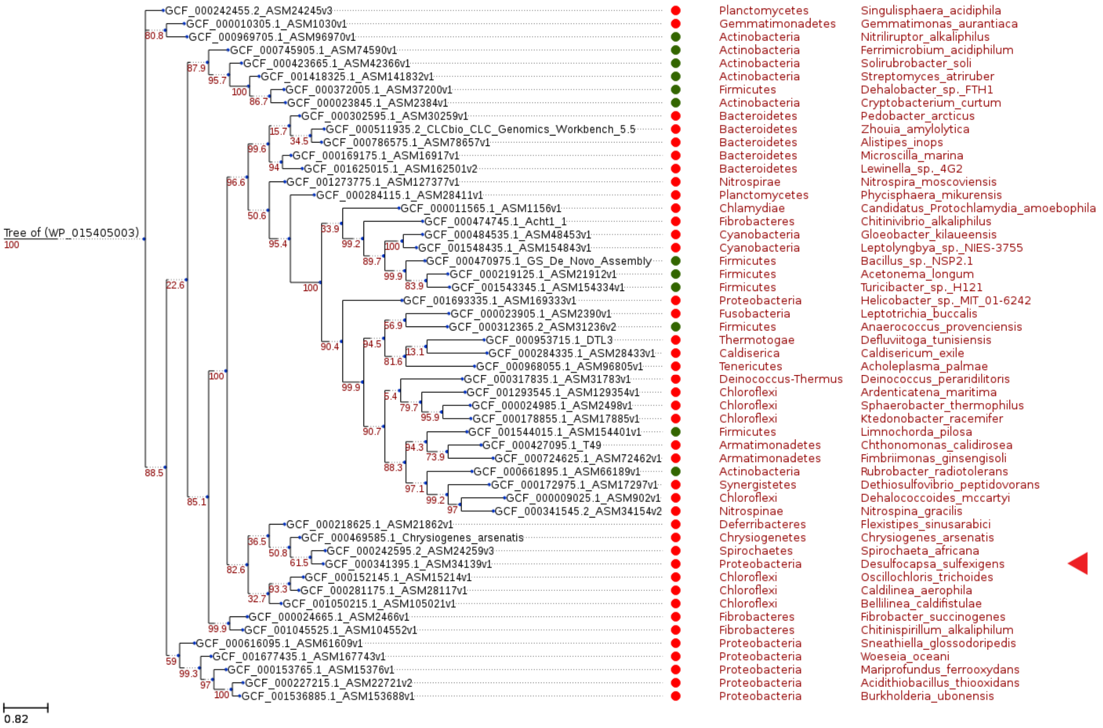

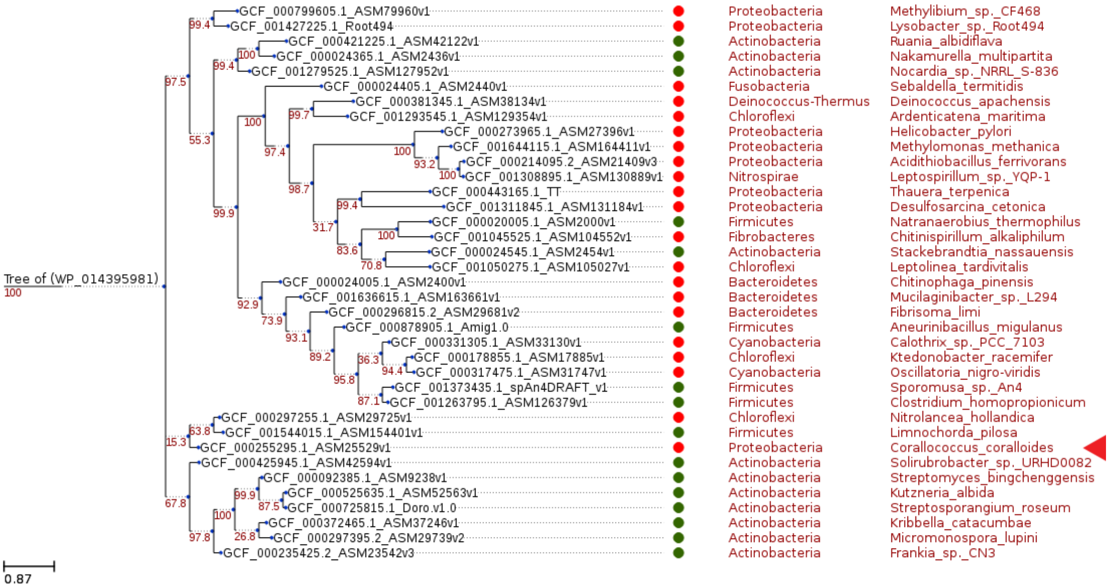
Tree of (WP_014395981)
100
GCF_000799605.1_ASM79960v1 Proteobacteria Methylibium_sp._CF468
GCF_001427225.1_Root494 Proteobacteria Lysobacter_sp._Root494
GCF_000421225.1_ASM42122v1 Actinobacteria Ruania_albidiflava
GCF_000024365.1_ASM2436v1 Actinobacteria Nakamurella_multipartita
GCF_001279525.1_ASM127952v1 Actinobacteria Nocardia_sp._NRRL_S-836
GCF_000024405.1_ASM2440v1 Fusobacteria Sebaldella_termitidis
GCF_000381345.1_ASM38134v1 Deinococcus-Thermus Deinococcus_apachensis
GCF_001293545.1_ASM129354v1 Chloroflexi Ardenticatena_maritima
GCF_000273965.1_ASM27396v1 Proteobacteria Helicobacter_pylori
GCF_001644115.1_ASM164411v1 Proteobacteria Methylomonas_methanica
GCF_000214095.2_ASM21409v3 Proteobacteria Acidithiobacillus_ferrivorans
GCF_001308895.1_ASM130889v1 Nitrospirae Leptospirillum_sp._YQP-1
GCF_000443165.1_TT Proteobacteria Thauera_terpenica
GCF_001311845.1_ASM131184v1 Proteobacteria Desulfosarcina_cetonica
GCF_000020005.1_ASM2000v1 Firmicutes Natranaerobius_thermophilus
GCF_001045525.1_ASM104552v1 Fibrobacteres Chitinispirillum_alkaliphilum
GCF_000024545.1_ASM2454v1 Actinobacteria Stackebrandtia_nassauensis
GCF_001050275.1_ASM105027v1 Chloroflexi Leptolinea_tardivitalis
GCF_000024005.1_ASM2400v1 Bacteroidetes Chitinophaga_pinensis
GCF_001636615.1_ASM163661v1 Bacteroidetes Mucilaginibacter_sp._L294
GCF_000296815.2_ASM29681v2 Bacteroidetes Fibrisoma_limi
GCF_000878905.1_Amig1.0 Firmicutes Aneurinibacillus_migulanus
GCF_000331305.1_ASM33130v1 Cyanobacteria Calothrix_sp._PCC_7103
GCF_000178855.1_ASM17885v1 Chloroflexi Ktedonobacter_racemifer
GCF_000317475.1_ASM31747v1 Cyanobacteria Oscillatoria_nigro-viridis
GCF_001373435.1_spAn4DRAFT_v1 Firmicutes Sporomusa_sp._An4
GCF_001263795.1_ASM126379v1 Firmicutes Clostridium_homopropionicum
GCF_000297255.1_ASM29725v1 Chloroflexi Nitrolancea_hollandica
GCF_001544015.1_ASM154401v1 Firmicutes Limnochorda_pilosa
GCF_000255295.1_ASM25529v1 Proteobacteria Corallococcus_coralloides
GCF_000425945.1_ASM42594v1 Actinobacteria Solirubrobacter_sp._URHD0082
GCF_000092385.1_ASM9238v1 Actinobacteria Streptomyces_bingchenggensis
GCF_000525635.1_ASM52563v1 Actinobacteria Kutzneria_albida
GCF_000725815.1_Doro.v1.0 Actinobacteria Streptosporangium_roseum
GCF_000372465.1_ASM37246v1 Actinobacteria Kribbella_catacumbae
GCF_000297395.2_ASM29739v2 Actinobacteria Micromonospora_lupini
GCF_000235425.2_ASM23542v3 Actinobacteria Frankia_sp._CN3
0.87

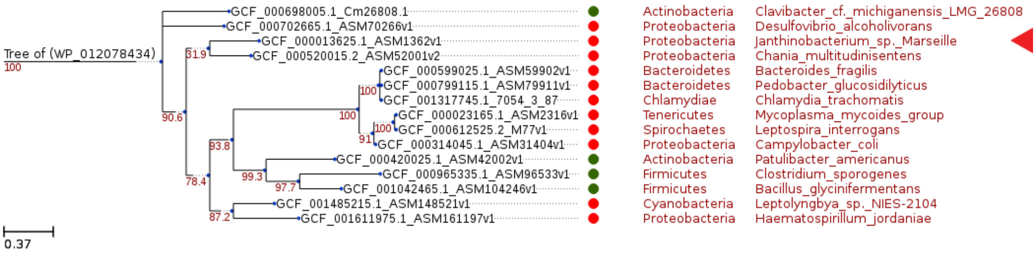
Tree of (WP_012078434)
100
GCF_000698005.1_Cm26808.1 Actinobacteria Clavibacter_cf._michiganensis_LMG_26808
GCF_000702665.1_ASM70266v1 Proteobacteria Desulfovibrio_alcoholivorans
GCF_000013625.1_ASM1362v1 Proteobacteria Janthinobacterium_sp._Marseille
GCF_000520015.2_ASM52001v2 Proteobacteria Chania_multitudinisentens
GCF_000599025.1_ASM59902v1 Bacteroidetes Bacteroides_fragilis
GCF_000799115.1_ASM79911v1 Bacteroidetes Pedobacter_glucosidilyticus
GCF_001317745.1_7054_3_87 Chlamydiae Chlamydia_trachomatis
GCF_000023165.1_ASM2316v1 Tenericutes Mycoplasma_mycoides_group
GCF_000612525.2_M77v1 Spirochaetes Leptospira_interrogans
GCF_000314045.1_ASM31404v1 Proteobacteria Campylobacter_coli
GCF_000420025.1_ASM42002v1 Actinobacteria Patulibacter_americanus
GCF_000965335.1_ASM96533v1 Firmicutes Clostridium_sporogenes
GCF_001042465.1_ASM104246v1 Firmicutes Bacillus_glycinifermentans
GCF_001485215.1_ASM148521v1 Cyanobacteria Leptolyngbya_sp._NIES-2104
GCF_001611975.1_ASM161197v1 Proteobacteria Haematospirillum_jordaniae
0.37

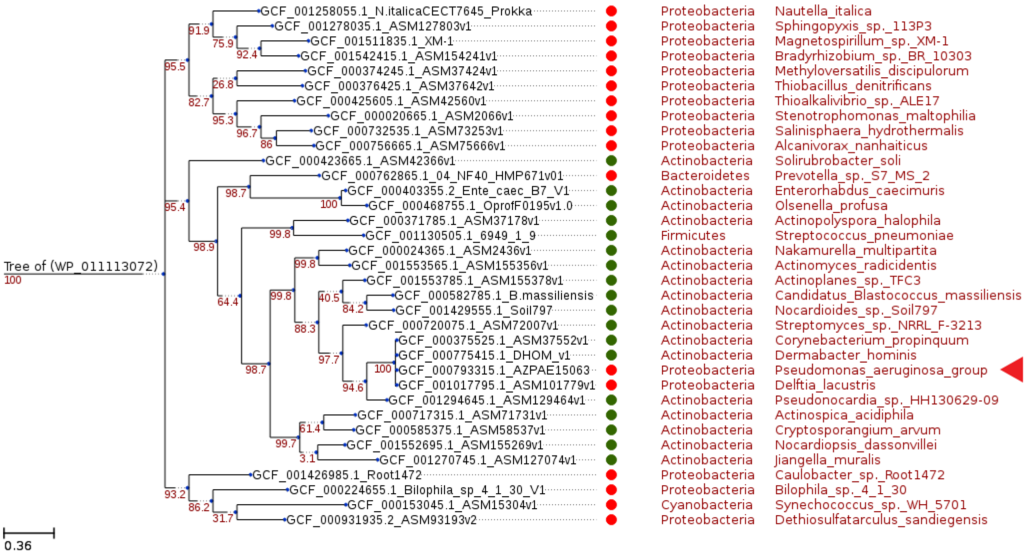
Tree of (WP_011113072)
100
GCF_001258055.1_N.italicaCECT7645_Prokka Proteobacteria Nautella_italica
GCF_001278035.1_ASM127803v1 Proteobacteria Sphingopyxis_sp._113P3
GCF_001511835.1_XM-1 Proteobacteria Magnetospirillum_sp._XM-1
GCF_001542415.1_ASM154241v1 Proteobacteria Bradyrhizobium_sp._BR_10303
GCF_000374245.1_ASM37424v1 Proteobacteria Methyloversatilis_discipulorum
GCF_000376425.1_ASM37642v1 Proteobacteria Thiobacillus_denitrificans
GCF_000425605.1_ASM42560v1 Proteobacteria Thioalkalivibrio_sp._ALE17
GCF_000020665.1_ASM2066v1 Proteobacteria Stenotrophomonas_maltophilia
GCF_000732535.1_ASM73253v1 Proteobacteria Salinisphaera_hydrothermalis
GCF_000756665.1_ASM75666v1 Proteobacteria Alcanivorax_nanhaiticus
GCF_000423665.1_ASM42366v1 Actinobacteria Solirubrobacter_soli
GCF_000762865.1_04_NF40_HMP671v01 Bacteroidetes Prevotella_sp._S7_MS_2
GCF_000403355.2_Ente_caec_B7_V1 Actinobacteria Enterorhabdus_caecimuris
GCF_000468755.1_OprofF0195v1.0 Actinobacteria Olsenella_profusa
GCF_000371785.1_ASM37178v1 Actinobacteria Actinopolyspora_halophila
GCF_001130505.1_6949_1_9 Firmicutes Streptococcus_pneumoniae
GCF_000024365.1_ASM2436v1 Actinobacteria Nakamurella_multipartita
GCF_001553565.1_ASM155356v1 Actinobacteria Actinomyces_radicidentis
GCF_001553785.1_ASM155378v1 Actinobacteria Actinoplanes_sp._TFC3
GCF_000582785.1_B.massiliensis Actinobacteria Candidatus_Blastococcus_massiliensis
GCF_001429555.1_Soil797 Actinobacteria Nocardioides_sp._Soil797
GCF_000720075.1_ASM72007v1 Actinobacteria Streptomyces_sp._NRRL_F-3213
GCF_000375525.1_ASM37552v1 Actinobacteria Corynebacterium_propinquum
GCF_000775415.1_DHOM_v1 Actinobacteria Dermabacter_hominis
GCF_000793315.1_AZPAE15063 Proteobacteria Pseudomonas_aeruginosa_group
GCF_001017795.1_ASM101779v1 Proteobacteria Delftia_lacustris
GCF_001294645.1_ASM129464v1 Actinobacteria Pseudonocardia_sp._HH130629-09
GCF_000717315.1_ASM71731v1 Actinobacteria Actinospica_acidiphila
GCF_000585375.1_ASM58537v1 Actinobacteria Cryptosporangium_arvum
GCF_001552695.1_ASM155269v1 Actinobacteria Nocardiopsis_dassonvillei
GCF_001270745.1_ASM127074v1 Actinobacteria Jiangella_muralis
GCF_001426985.1_Root1472 Proteobacteria Caulobacter_sp._Root1472
GCF_000224655.1_Bilophila_sp_4_1_30_V1 Proteobacteria Bilophila_sp._4_1_30
GCF_000153045.1_ASM15304v1 Cyanobacteria Synechococcus_sp._WH_5701
GCF_000931935.2_ASM93193v2 Proteobacteria Dethiosulfatarculus_sandiegensis
0.36

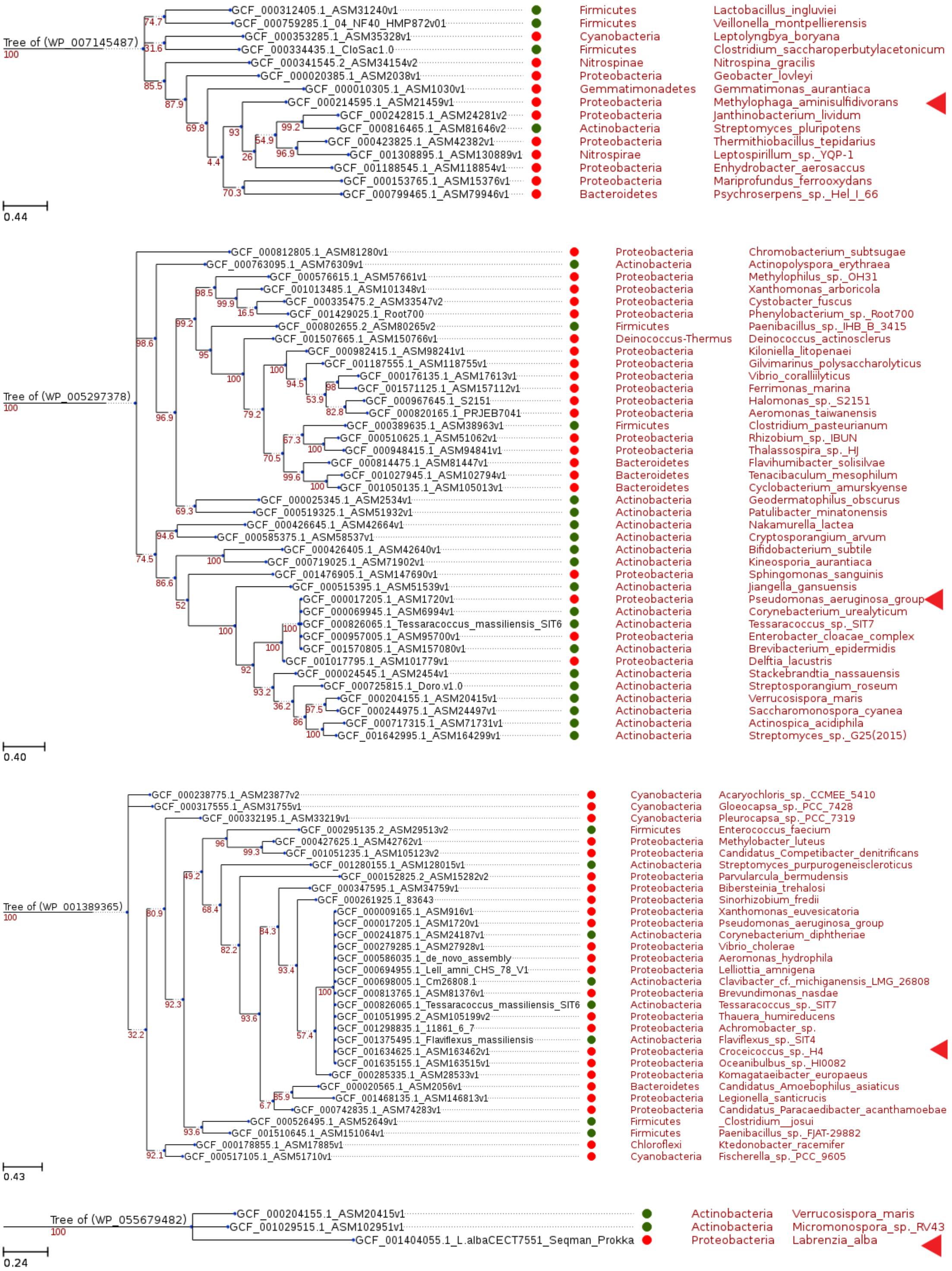

Tree of (WP_007145487)
GCF_000312405.1_ASM31240v1 Firmicutes Lactobacillus_ingluviei
GCF_000759285.1_04_NF40_HMP872v01 Firmicutes Veillonella_montpellierensis
GCF_000353285.1_ASM35328v1 Cyanobacteria Leptolyngbya_boryana
GCF_000334435.1_CloSac1.0 Firmicutes Clostridium_saccharoperbutylacetonicum
GCF_000341545.2_ASM34154v2 Nitrospinae Nitrospina_gracilis
GCF_000020385.1_ASM2038v1 Proteobacteria Geobacter_lovleyi
GCF_000010305.1_ASM1030v1 Gemmatimonadetes Gemmatimonas_aurantiaca
GCF_000214595.1_ASM21459v1 Proteobacteria Methylophaga_aminisulfidivorans
GCF_000242815.1_ASM24281v2 Proteobacteria Janthinobacterium_lividum
GCF_000816465.1_ASM81646v2 Actinobacteria Streptomyces_pluripotens
GCF_000423825.1_ASM42382v1 Proteobacteria Thermithiobacillus_tepidarius
GCF_001308895.1_ASM130889v1 Nitrospirae Leptospirillum_sp._YQP-1
GCF_001188545.1_ASM118854v1 Proteobacteria Enhydrobacter_aerosaccus
GCF_000153765.1_ASM15376v1 Proteobacteria Mariprofundus_ferrooxydans
GCF_000799465.1_ASM79946v1 Bacteroidetes Psychroserpens_sp._Hel_I_66
0.44
Tree of (WP_005297378)
GCF_000812805.1_ASM81280v1 Proteobacteria Chromobacterium_subtsugae
GCF_000763095.1_ASM76309v1 Actinobacteria Actinopolyspora_erythraea
GCF_000576615.1_ASM57661v1 Proteobacteria Methylophilus_sp._OH31
GCF_001013485.1_ASM101348v1 Proteobacteria Xanthomonas_arboricola
GCF_000335475.2_ASM33547v2 Proteobacteria Cystobacter_fuscus
GCF_001429025.1_Root700 Proteobacteria Phenylobacterium_sp._Root700
GCF_000802655.2_ASM80265v2 Firmicutes Paenibacillus_sp._IHB_B_3415
GCF_001507665.1_ASM150766v1 Deinococcus-Thermus Deinococcus_actinosclerus
GCF_000982415.1_ASM98241v1 Proteobacteria Kiloniella_litopenaei
GCF_001187555.1_ASM118755v1 Proteobacteria Gilvimarinus_polysaccharolyticus
GCF_000176135.1_ASM17613v1 Proteobacteria Vibrio_coralliilyticus
GCF_001571125.1_ASM157112v1 Proteobacteria Ferrimonas_marina
GCF_000967645.1_S2151 Proteobacteria Halomonas_sp._S2151
GCF_000820165.1_PRJEB7041 Proteobacteria Aeromonas_taiwanensis
GCF_000389635.1_ASM38963v1 Firmicutes Clostridium_pasteurianum
GCF_000510625.1_ASM51062v1 Proteobacteria Rhizobium_sp._IBUN
GCF_000948415.1_ASM94841v1 Proteobacteria Thalassospira_sp._HJ
GCF_000814475.1_ASM81447v1 Bacteroidetes Flavihumibacter_solisilvae
GCF_001027945.1_ASM102794v1 Bacteroidetes Tenacibaculum_mesophilum
GCF_001050135.1_ASM105013v1 Bacteroidetes Cyclobacterium_amurskyense
GCF_000025345.1_ASM2534v1 Actinobacteria Geodermatophilus_obscurus
GCF_000519325.1_ASM51932v1 Actinobacteria Patulibacter_minatonensis
GCF_000426645.1_ASM42664v1 Actinobacteria Nakamurella_lactea
GCF_000585375.1_ASM58537v1 Actinobacteria Cryptosporangium_arvum
GCF_000426405.1_ASM42640v1 Actinobacteria Bifidobacterium_subtile
GCF_000719025.1_ASM71902v1 Actinobacteria Kineosporia_aurantiaca
GCF_001476905.1_ASM147690v1 Proteobacteria Sphingomonas_sanguinis
GCF_000515395.1_ASM51539v1 Actinobacteria Jiangella_gansuensis
GCF_000017205.1_ASM1720v1 Proteobacteria Pseudomonas_aeruginosa_group
GCF_000069945.1_ASM6994v1 Actinobacteria Corynebacterium_urealyticum
GCF_000826065.1_Tessaracoccus_massiliensis_SIT6 Actinobacteria Tessaracoccus_sp._SIT7
GCF_000957005.1_ASM95700v1 Proteobacteria Enterobacter_cloacae_complex
GCF_001570805.1_ASM157080v1 Actinobacteria Brevibacterium_epidermidis
GCF_001017795.1_ASM101779v1 Proteobacteria Delftia_lacustris
GCF_000024545.1_ASM2454v1 Actinobacteria Stackebrandtia_nassauensis
GCF_000725815.1_Doro.v1.0 Actinobacteria Streptosporangium_roseum
GCF_000204155.1_ASM20415v1 Actinobacteria Verrucosispora_maris
GCF_000244975.1_ASM24497v1 Actinobacteria Saccharomonospora_cyanea
GCF_000717315.1_ASM71731v1 Actinobacteria Actinospica_acidiphila
GCF_001642995.1_ASM164299v1 Actinobacteria Streptomyces_sp._G25(2015)
0.40
Tree of (WP_001389365)
GCF_000238775.1_ASM23877v2 Cyanobacteria Acaryochloris_sp._CCMEE_5410
GCF_000317555.1_ASM31755v1 Cyanobacteria Gloeocapsa_sp._PCC_7428
GCF_000332195.1_ASM33219v1 Cyanobacteria Pleurocapsa_sp._PCC_7319
GCF_000295135.2_ASM29513v2 Firmicutes Enterococcus_faecium
GCF_000427625.1_ASM42762v1 Proteobacteria Methylobacter_luteus
GCF_001051235.1_ASM105123v2 Proteobacteria Candidatus_Competibacter_denitrificans
GCF_001280155.1_ASM128015v1 Actinobacteria Streptomyces_purpurogeneiscleroticus
GCF_000152825.2_ASM15282v2 Proteobacteria Parvularcula_bermudensis
GCF_000347595.1_ASM34759v1 Proteobacteria Bibersteinia_trehalosi
GCF_000261925.1_83643 Proteobacteria Sinorhizobium_fredii
GCF_000009165.1_ASM916v1 Proteobacteria Xanthomonas_euvesicatoria
GCF_000017205.1_ASM1720v1 Proteobacteria Pseudomonas_aeruginosa_group
GCF_000241875.1_ASM24187v1 Actinobacteria Corynebacterium_diphtheriae
GCF_000279285.1_ASM27928v1 Proteobacteria Vibrio_cholerae
GCF_000586035.1_de_novo_assembly Proteobacteria Aeromonas_hydrophila
GCF_000694955.1_Leli_amni_CHS_78_V1 Proteobacteria Lelliottia_amnigena
GCF_000698005.1_Cm26808.1 Actinobacteria Clavibacter_cf._michiganensis_LMG_26808
GCF_000813765.1_ASM81376v1 Proteobacteria Brevundimonas_nasdae
GCF_000826065.1_Tessaracoccus_massiliensis_SIT6 Actinobacteria Tessaracoccus_sp._SIT7
GCF_001051995.2_ASM105199v2 Proteobacteria Thauera_humireducens
GCF_001298835.1_11861_6_7 Proteobacteria Achromobacter_sp.
GCF_001375495.1_Flaviflexus_massiliensis Actinobacteria Flaviflexus_sp._SIT4
GCF_001634625.1_ASM163462v1 Proteobacteria Croceicoccus_sp._H4
GCF_001635155.1_ASM163515v1 Proteobacteria Oceanibulbus_sp._HI0082
GCF_000285335.1_ASM28533v1 Proteobacteria Komagataeibacter_europaeus
GCF_000020565.1_ASM2056v1 Bacteroidetes Candidatus_Amoebophilus_asiaticus
GCF_001468135.1_ASM146813v1 Proteobacteria Legionella_santicrucis
GCF_000742835.1_ASM74283v1 Proteobacteria Candidatus_Paracaedibacter_acanthamoebae
GCF_000526495.1_ASM52649v1 Firmicutes _Clostridium__josui
GCF_001510645.1_ASM151064v1 Firmicutes Paenibacillus_sp._FJAT-29882
GCF_000178855.1_ASM17885v1 Chloroflexi Ktedonobacter_racemifer
GCF_000517105.1_ASM51710v1 Cyanobacteria Fischerella_sp._PCC_9605
0.43
Tree of (WP_055679482)
GCF_000204155.1_ASM20415v1 Actinobacteria Verrucosispora_maris
GCF_001029515.1_ASM102951v1 Actinobacteria Micromonospora_sp._RV43
GCF_001404055.1_L.albaCECT7551_Seqman_Prokka Proteobacteria Labrenzia_alba
0.24

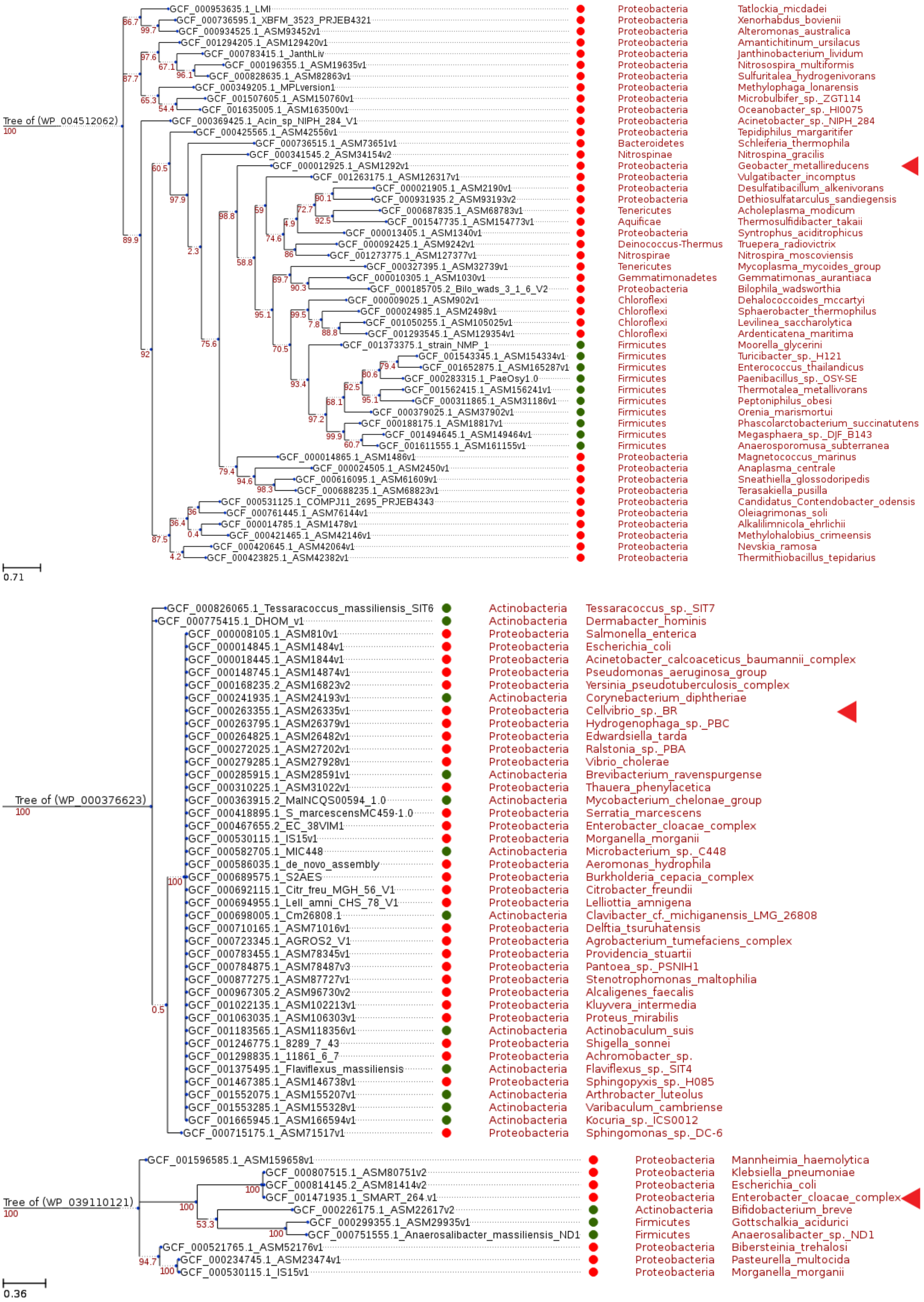
Tree of (WP_004512062)
GCF_000953635.1_LMI Proteobacteria Tatlockia_micdadei
GCF_000736595.1_XBFM_3523_PRJEB4321 Proteobacteria Xenorhabdus_bovienii
GCF_000934525.1_ASM93452v1 Proteobacteria Alteromonas_australica
GCF_001294205.1_ASM129420v1 Proteobacteria Amantichitinum_ursilacus
GCF_000783415.1_JanthLiv Proteobacteria Janthinobacterium_lividum
GCF_000196355.1_ASM19635v1 Proteobacteria Nitrosospira_multiformis
GCF_000828635.1_ASM82863v1 Proteobacteria Sulfuritalea_hydrogenivorans
GCF_000349205.1_MPLversion1 Proteobacteria Methylophaga_lonarensis
GCF_001507605.1_ASM150760v1 Proteobacteria Microbulbifer_sp._ZGT114
GCF_001635005.1_ASM163500v1 Proteobacteria Oceanobacter_sp._HI0075
GCF_000369425.1_Acin_sp_NIPH_284_V1 Proteobacteria Acinetobacter_sp._NIPH_284
GCF_000425565.1_ASM42556v1 Proteobacteria Tepidiphilus_margaritifer
GCF_000736515.1_ASM73651v1 Bacteroidetes Schleiferia_thermophila
GCF_000341545.2_ASM34154v2 Nitrospinae Nitrospina_gracilis
GCF_000012925.1_ASM1292v1 Proteobacteria Geobacter_metallireducens
GCF_001263175.1_ASM126317v1 Proteobacteria Vulgatibacter_incomptus
GCF_000021905.1_ASM2190v1 Proteobacteria Desulfatibacillum_alkenivorans
GCF_000931935.2_ASM93193v2 Proteobacteria Dethiosulfatarculus_sandiegensis
GCF_000687835.1_ASM68783v1 Tenericutes Acholeplasma_modicum
GCF_001547735.1_ASM154773v1 Aquificae Thermosulfidibacter_takaii
GCF_000013405.1_ASM1340v1 Proteobacteria Syntrophus_aciditrophicus
GCF_000092425.1_ASM9242v1 Deinococcus-Thermus Truepera_radiovictrix
GCF_001273775.1_ASM127377v1 Nitrospirae Nitrospira_moscoviensis
GCF_000327395.1_ASM32739v1 Tenericutes Mycoplasma_mycoides_group
GCF_000010305.1_ASM1030v1 Gemmatimonadetes Gemmatimonas_aurantiaca
GCF_000185705.2_Bilo_wads_3_1_6_V2 Proteobacteria Bilophila_wadsworthia
GCF_000009025.1_ASM902v1 Chloroflexi Dehalococcoides_mccartyi
GCF_000024985.1_ASM2498v1 Chloroflexi Sphaerobacter_thermophilus
GCF_001050255.1_ASM105025v1 Chloroflexi Levilinea_saccharolytica
GCF_001293545.1_ASM129354v1 Chloroflexi Ardenticatena_maritima
GCF_001373375.1_strain_NMP_1 Firmicutes Moorella_glycerini
GCF_001543345.1_ASM154334v1 Firmicutes Turicibacter_sp._H121
GCF_001652875.1_ASM165287v1 Firmicutes Enterococcus_thailandicus
GCF_000283315.1_PaeOsy1.0 Firmicutes Paenibacillus_sp._OSY-SE
GCF_001562415.1_ASM156241v1 Firmicutes Thermotalea_metallivorans
GCF_000311865.1_ASM31186v1 Firmicutes Peptoniphilus_obesi
GCF_000379025.1_ASM37902v1 Firmicutes Orenia_marismortui
GCF_000188175.1_ASM18817v1 Firmicutes Phascolarctobacterium_succinatutens
GCF_001494645.1_ASM149464v1 Firmicutes Megasphaera_sp._DJF_B143
GCF_001611555.1_ASM161155v1 Firmicutes Anaerosporomusa_subterranea
GCF_000014865.1_ASM1486v1 Proteobacteria Magnetococcus_marinus
GCF_000024505.1_ASM2450v1 Proteobacteria Anaplasma_centrale
GCF_000616095.1_ASM61609v1 Proteobacteria Sneathiella_glossodoripedis
GCF_000688235.1_ASM68823v1 Proteobacteria Terasakiella_pusilla
GCF_000531125.1_COMPJ11_2695_PRJEB4343 Proteobacteria Candidatus_Contendobacter_odensis
GCF_000761445.1_ASM76144v1 Proteobacteria Oleiagrimonas_soli
GCF_000014785.1_ASM1478v1 Proteobacteria Alkalilimnicola_ehrlichii
GCF_000421465.1_ASM42146v1 Proteobacteria Methylohalobius_crimeensis
GCF_000420645.1_ASM42064v1 Proteobacteria Nevskia_ramosa
GCF_000423825.1_ASM42382v1 Proteobacteria Thermithiobacillus_tepidarius
0.71
Tree of (WP_000376623)
GCF_000826065.1_Tessaracoccus_massiliensis_SIT6 Actinobacteria Tessaracoccus_sp._SIT7
GCF_000775415.1_DHOM_v1 Actinobacteria Dermabacter_hominis
GCF_000008105.1_ASM810v1 Proteobacteria Salmonella_enterica
GCF_000014845.1_ASM1484v1 Proteobacteria Escherichia_coli
GCF_000018445.1_ASM1844v1 Proteobacteria Acinetobacter_calcoaceticus_baumannii_complex
GCF_000148745.1_ASM14874v1 Proteobacteria Pseudomonas_aeruginosa_group
GCF_000168235.2_ASM16823v2 Proteobacteria Yersinia_pseudotuberculosis_complex
GCF_000241935.1_ASM24193v1 Actinobacteria Corynebacterium_diphtheriae
GCF_000263355.1_ASM26335v1 Proteobacteria Cellvibrio_sp._BR
GCF_000263795.1_ASM26379v1 Proteobacteria Hydrogenophaga_sp._PBC
GCF_000264825.1_ASM26482v1 Proteobacteria Edwardsiella_tarda
GCF_000272025.1_ASM27202v1 Proteobacteria Ralstonia_sp._PBA
GCF_000279285.1_ASM27928v1 Proteobacteria Vibrio_cholerae
GCF_000285915.1_ASM28591v1 Actinobacteria Brevibacterium_ravenspurgense
GCF_000310225.1_ASM31022v1 Proteobacteria Thauera_phenylacetica
GCF_000363915.2_MalNCQS00594_1.0 Actinobacteria Mycobacterium_chelonae_group
GCF_000418895.1_S_marcescensMC459-1.0 Proteobacteria Serratia_marcescens
GCF_000467655.2_EC_38VIM1 Proteobacteria Enterobacter_cloacae_complex
GCF_000530115.1_IS15v1 Proteobacteria Morganella_morganii
GCF_000582705.1_MIC448 Actinobacteria Microbacterium_sp._C448
GCF_000586035.1_de_novo_assembly Proteobacteria Aeromonas_hydrophila
GCF_000689575.1_S2AES Proteobacteria Burkholderia_cepacia_complex
GCF_000692115.1_Citr_freu_MGH_56_V1 Proteobacteria Citrobacter_freundii
GCF_000694955.1_Lell_amni_CHS_78_V1 Proteobacteria Lelliottia_amnigena
GCF_000698005.1_Cm26808.1 Actinobacteria Clavibacter_cf._michiganensis_LMG_26808
GCF_000710165.1_ASM71016v1 Proteobacteria Delftia_tsuruhatensis
GCF_000723345.1_AGROS2_V1 Proteobacteria Agrobacterium_tumefaciens_complex
GCF_000783455.1_ASM78345v1 Proteobacteria Providencia_stuartii
GCF_000784875.1_ASM78487v3 Proteobacteria Pantoea_sp._PSNIH1
GCF_000877275.1_ASM87727v1 Proteobacteria Stenotrophomonas_maltophilia
GCF_000967305.2_ASM96730v2 Proteobacteria Alcaligenes_faecalis
GCF_001022135.1_ASM102213v1 Proteobacteria Kluyvera_intermedia
GCF_001063035.1_ASM106303v1 Proteobacteria Proteus_mirabilis
GCF_001183565.1_ASM118356v1 Actinobacteria Actinobaculum_suis
GCF_001246775.1_8289_7_43 Proteobacteria Shigella_sonnei
GCF_001298835.1_11861_6_7 Proteobacteria Achromobacter_sp.
GCF_001375495.1_Flaviflexus_massiliensis Actinobacteria Flaviflexus_sp._SIT4
GCF_001467385.1_ASM146738v1 Proteobacteria Sphingopyxis_sp._H085
GCF_001552075.1_ASM155207v1 Actinobacteria Arthrobacter_luteolus
GCF_001553285.1_ASM155328v1 Actinobacteria Varibaculum_cambriense
GCF_001665945.1_ASM166594v1 Actinobacteria Kocuria_sp._ICS0012
GCF_000715175.1_ASM71517v1 Proteobacteria Sphingomonas_sp._DC-6
Tree of (WP_039110121)
GCF_001596585.1_ASM159658v1 Proteobacteria Mannheimia_haemolytica
GCF_000807515.1_ASM80751v2 Proteobacteria Klebsiella_pneumoniae
GCF_000814145.2_ASM81414v2 Proteobacteria Escherichia_coli
GCF_001471935.1_SMART_264.v1 Proteobacteria Enterobacter_cloacae_complex
GCF_000226175.1_ASM22617v2 Actinobacteria Bifidobacterium_breve
GCF_000299355.1_ASM29935v1 Firmicutes Gottschalkia_acidurici
GCF_000751555.1_Anaerosalibacter_massiliensis_ND1 Firmicutes Anaerosalibacter_sp._ND1
GCF_000521765.1_ASM52176v1 Proteobacteria Bibersteinia_trehalosi
GCF_000234745.1_ASM23474v1 Proteobacteria Pasteurella_multocida
GCF_000530115.1_IS15v1 Proteobacteria Morganella_morganii
0.36

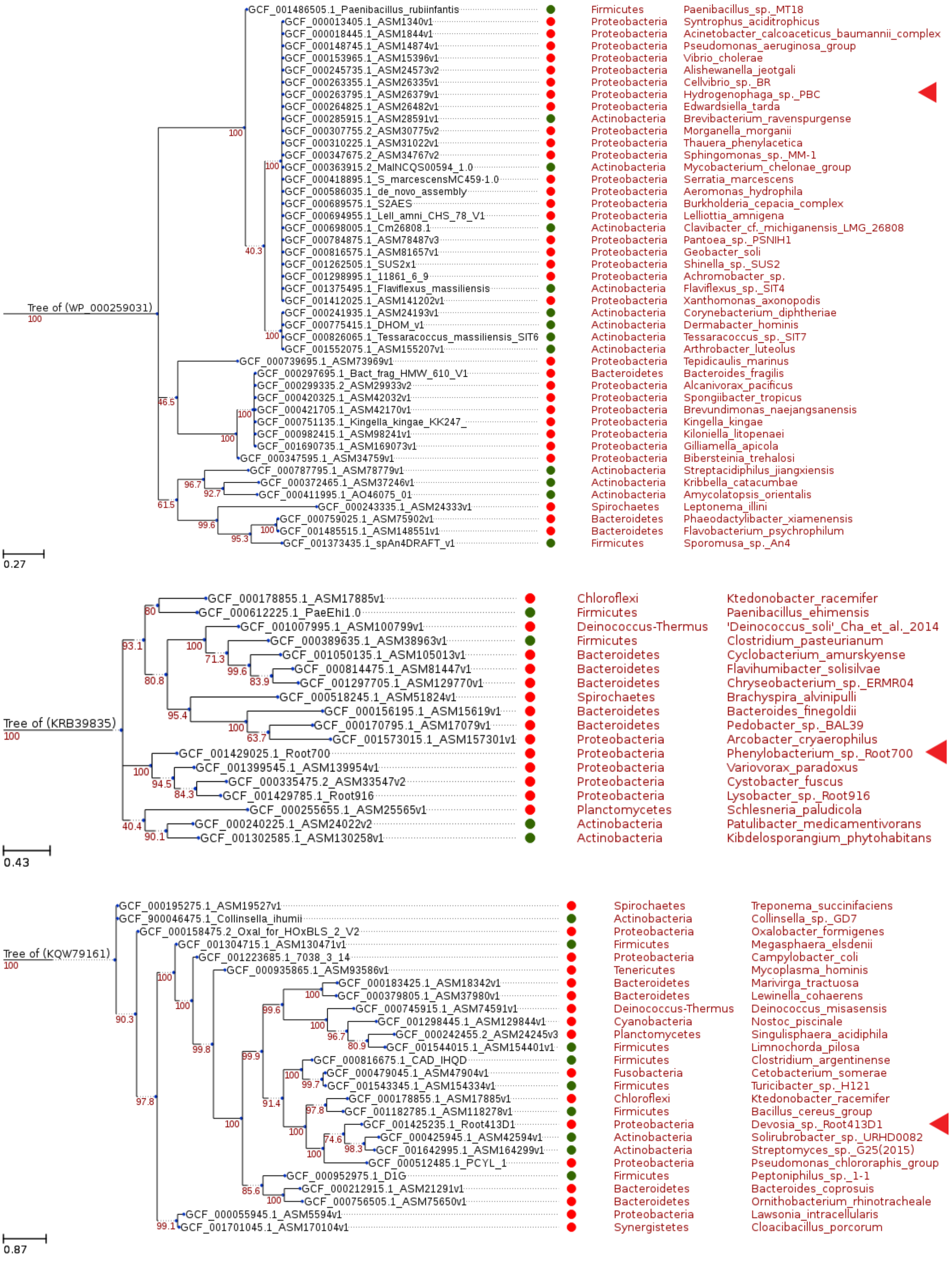

Tree of (WP_000259031)
GCF_001486505.1_Paenibacillus_rubiinfantis Firmicutes Paenibacillus_sp._MT18
GCF_000013405.1_ASM1340v1 Proteobacteria Syntrophus_aciditrophicus
GCF_000018445.1_ASM1844v1 Proteobacteria Acinetobacter_calcoaceticus_baumannii_complex
GCF_000148745.1_ASM14874v1 Proteobacteria Pseudomonas_aeruginosa_group
GCF_000153965.1_ASM15396v1 Proteobacteria Vibrio_cholerae
GCF_000245735.1_ASM24573v2 Proteobacteria Alishewanella_jeotgali
GCF_000263355.1_ASM26335v1 Proteobacteria Cellvibrio_sp._BR
GCF_000263795.1_ASM26379v1 Proteobacteria Hydrogenophaga_sp._PBC
GCF_000264825.1_ASM26482v1 Proteobacteria Edwardsiella_tarda
GCF_000285915.1_ASM28591v1 Actinobacteria Brevibacterium_ravenspurgense
GCF_000307755.2_ASM30775v2 Proteobacteria Morganella_morganii
GCF_000310225.1_ASM31022v1 Proteobacteria Thauera_phenylacetica
GCF_000347675.2_ASM34767v2 Proteobacteria Sphingomonas_sp._MM-1
GCF_000363915.2_MalNCQS00594_1.0 Actinobacteria Mycobacterium_chelonae_group
GCF_000418895.1_S_marcescensMC459-1.0 Proteobacteria Serratia_marcescens
GCF_000586035.1_de_novo_assembly Proteobacteria Aeromonas_hydrophila
GCF_000689575.1_S2AES Proteobacteria Burkholderia_cepacia_complex
GCF_000694955.1_Lell_amni_CHS_78_V1 Proteobacteria Lelliottia_amnigena
GCF_000698005.1_Cm26808.1 Actinobacteria Clavibacter_cf._michiganensis_LMG_26808
GCF_000784875.1_ASM78487v3 Proteobacteria Pantoea_sp._PSNIH1
GCF_000816575.1_ASM81657v1 Proteobacteria Geobacter_soli
GCF_001262505.1_SUS2x1 Proteobacteria Shinella_sp._SUS2
GCF_001298995.1_11861_6_9 Proteobacteria Achromobacter_sp.
GCF_001375495.1_Flaviflexus_massiliensis Actinobacteria Flaviflexus_sp._SIT4
GCF_001412025.1_ASM141202v1 Proteobacteria Xanthomonas_axonopodis
GCF_000241935.1_ASM24193v1 Actinobacteria Corynebacterium_diphtheriae
GCF_000775415.1_DHOM_v1 Actinobacteria Dermabacter_hominis
GCF_000826065.1_Tessaracoccus_massiliensis_SIT6 Actinobacteria Tessaracoccus_sp._SIT7
GCF_001552075.1_ASM155207v1 Actinobacteria Arthrobacter_luteolus
GCF_000739695.1_ASM73969v1 Proteobacteria Tepidicaulis_marinus
GCF_000297695.1_Bact_frag_HMW_610_V1 Bacteroidetes Bacteroides_fragilis
GCF_000299335.2_ASM29933v2 Proteobacteria Alcanivorax_pacificus
GCF_000420325.1_ASM42032v1 Proteobacteria Spongiibacter_tropicus
GCF_000421705.1_ASM42170v1 Proteobacteria Brevundimonas_naejangsanensis
GCF_000751135.1_Kingella_kingae_KK247_ Proteobacteria Kingella_kingae
GCF_000982415.1_ASM98241v1 Proteobacteria Kiloniella_litopenaei
GCF_001690735.1_ASM169073v1 Proteobacteria Gilliamella_apicola
GCF_000347595.1_ASM34759v1 Proteobacteria Bibersteinia_trehalosi
GCF_000787795.1_ASM78779v1 Actinobacteria Streptacidiphilus_jiangxiensis
GCF_000372465.1_ASM37246v1 Actinobacteria Kribbella_catacumbae
GCF_000411995.1_AO46075_01 Actinobacteria Amycolatopsis_orientalis
GCF_000243335.1_ASM24333v1 Spirochaetes Leptonema_illini
GCF_000759025.1_ASM75902v1 Bacteroidetes Phaeodactylibacter_xiamenensis
GCF_001485515.1_ASM148551v1 Bacteroidetes Flavobacterium_psychrophilum
GCF_001373435.1_spAn4DRAFT_v1 Firmicutes Sporomusa_sp._An4
0.27
Tree of (KRB39835)
GCF_000178855.1_ASM17885v1 Chloroflexi Ktedonobacter_racemifer
GCF_000612225.1_PaeEhi1.0 Firmicutes Paenibacillus_ehimensis
GCF_001007995.1_ASM100799v1 Deinococcus-Thermus 'Deinococcus_soli'_Cha_et_al._2014
GCF_000389635.1_ASM38963v1 Firmicutes Clostridium_pasteurianum
GCF_001050135.1_ASM105013v1 Bacteroidetes Cyclobacterium_amurskyense
GCF_000814475.1_ASM81447v1 Bacteroidetes Flavihumibacter_solisilvae
GCF_001297705.1_ASM129770v1 Bacteroidetes Chryseobacterium_sp._ERMR04
GCF_000518245.1_ASM51824v1 Spirochaetes Brachyspira_alvinipulli
GCF_000156195.1_ASM15619v1 Bacteroidetes Bacteroides_finegoldii
GCF_000170795.1_ASM17079v1 Bacteroidetes Pedobacter_sp._BAL39
GCF_001573015.1_ASM157301v1 Proteobacteria Arcobacter_cryaerophilus
GCF_001429025.1_Root700 Proteobacteria Phenylobacterium_sp._Root700
GCF_001399545.1_ASM139954v1 Proteobacteria Variovorax_paradoxus
GCF_000335475.2_ASM33547v2 Proteobacteria Cystobacter_fuscus
GCF_001429785.1_Root916 Proteobacteria Lysobacter_sp._Root916
GCF_000255655.1_ASM25565v1 Planctomycetes Schlesneria_paludicola
GCF_000240225.1_ASM24022v2 Actinobacteria Patulibacter_medicamentivorans
GCF_001302585.1_ASM130258v1 Actinobacteria Kibdelosporangium_phytohabitans
0.43
Tree of (KQW79161)
GCF_000195275.1_ASM19527v1 Spirochaetes Treponema_succinifaciens
GCF_900046475.1_Collinsella_ihumii Actinobacteria Collinsella_sp._GD7
GCF_000158475.2_Oxal_for_HOxBLS_2_V2 Proteobacteria Oxalobacter_formigenes
GCF_001304715.1_ASM130471v1 Firmicutes Megasphaera_elsdenii
GCF_001223685.1_7038_3_14 Proteobacteria Campylobacter_coli
GCF_000935865.1_ASM93586v1 Tenericutes Mycoplasma_hominis
GCF_000183425.1_ASM18342v1 Bacteroidetes Marivirga_tractuosa
GCF_000379805.1_ASM37980v1 Bacteroidetes Lewinella_cohaerens
GCF_000745915.1_ASM74591v1 Deinococcus-Thermus Deinococcus_misasensis
GCF_001298445.1_ASM129844v1 Cyanobacteria Nostoc_piscinale
GCF_000242455.2_ASM24245v3 Planctomycetes Singulisphaera_acidiphila
GCF_001544015.1_ASM154401v1 Firmicutes Limnochorda_pilosa
GCF_000816675.1_CAD_IHQD Firmicutes Clostridium_argentinense
GCF_000479045.1_ASM47904v1 Fusobacteria Cetobacterium_somerae
GCF_001543345.1_ASM154334v1 Firmicutes Turicibacter_sp._H121
GCF_000178855.1_ASM17885v1 Chloroflexi Ktedonobacter_racemifer
GCF_001182785.1_ASM118278v1 Firmicutes Bacillus_cereus_group
GCF_001425235.1_Root413D1 Proteobacteria Devosia_sp._Root413D1
GCF_000425945.1_ASM42594v1 Actinobacteria Solirubrobacter_sp._URHD0082
GCF_001642995.1_ASM164299v1 Actinobacteria Streptomyces_sp._G25(2015)
GCF_000512485.1_PCYL_1 Proteobacteria Pseudomonas_chlororaphis_group
GCF_000952975.1_D1G Firmicutes Peptoniphilus_sp._1-1
GCF_000212915.1_ASM21291v1 Bacteroidetes Bacteroides_coprosuis
GCF_000756505.1_ASM75650v1 Bacteroidetes Ornithobacterium_rhinotracheale
GCF_000055945.1_ASM5594v1 Proteobacteria Lawsonia_intracellularis
GCF_001701045.1_ASM170104v1 Synergistetes Cloacibacillus_porcorum
0.87

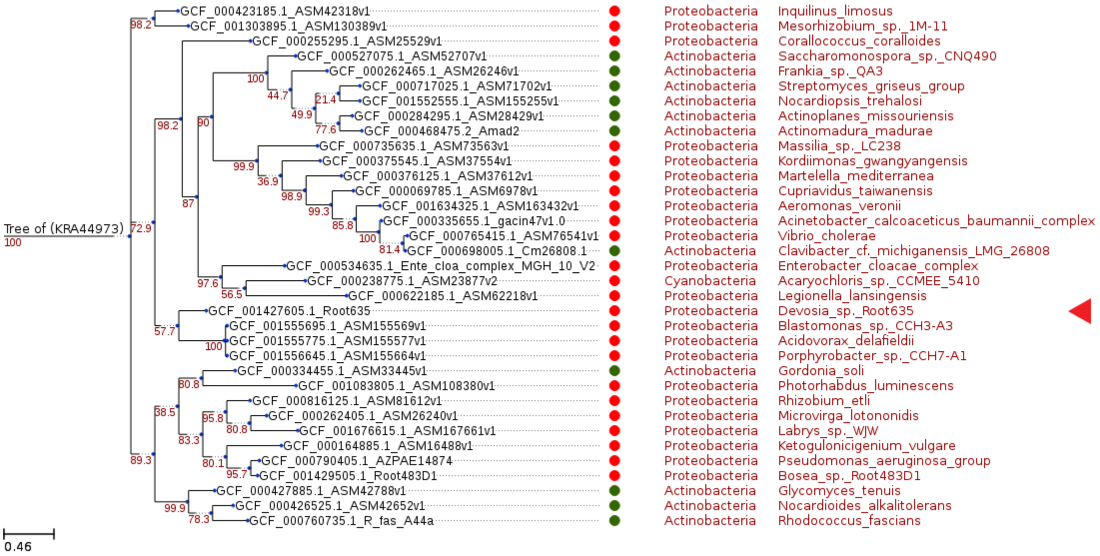
Tree of (KRA44973)
100
GCF_000423185.1_ASM42318v1 Proteobacteria Inquilinus_limosus
GCF_001303895.1_ASM130389v1 Proteobacteria Mesorhizobium_sp._1M-11
GCF_000255295.1_ASM25529v1 Proteobacteria Corallococcus_coralloides
GCF_000527075.1_ASM52707v1 Actinobacteria Saccharomonospora_sp._CNQ490
GCF_000262465.1_ASM26246v1 Actinobacteria Frankia_sp._QA3
GCF_000717025.1_ASM71702v1 Actinobacteria Streptomyces_griseus_group
GCF_001552555.1_ASM155255v1 Actinobacteria Nocardiopsis_trehalosi
GCF_000284295.1_ASM28429v1 Actinobacteria Actinoplanes_missouriensis
GCF_000468475.2_Amad2 Actinobacteria Actinomadura_madurae
GCF_000735635.1_ASM73563v1 Proteobacteria Massilia_sp._LC238
GCF_000375545.1_ASM37554v1 Proteobacteria Kordiimonas_gwangyangensis
GCF_000376125.1_ASM37612v1 Proteobacteria Martelella_mediterranea
GCF_000069785.1_ASM6978v1 Proteobacteria Cupriavidus_taiwanensis
GCF_001634325.1_ASM163432v1 Proteobacteria Aeromonas_veronii
GCF_000335655.1_gacin47v1.0 Proteobacteria Acinetobacter_calcoaceticus_baumannii_complex
GCF_000765415.1_ASM76541v1 Proteobacteria Vibrio_cholerae
GCF_000698005.1_Cm26808.1 Actinobacteria Clavibacter_cf._michiganensis_LMG_26808
GCF_000534635.1_Ente_cloa_complex_MGH_10_V2 Proteobacteria Enterobacter_cloacae_complex
GCF_000238775.1_ASM23877v2 Cyanobacteria Acaryochloris_sp._CCMEE_5410
GCF_000622185.1_ASM62218v1 Proteobacteria Legionella_lansingensis
GCF_001427605.1_Root635 Proteobacteria Devosia_sp._Root635
GCF_001555695.1_ASM155569v1 Proteobacteria Blastomonas_sp._CCH3-A3
GCF_001555775.1_ASM155577v1 Proteobacteria Acidovorax_delafieldii
GCF_001556645.1_ASM155664v1 Proteobacteria Porphyrobacter_sp._CCH7-A1
GCF_000334455.1_ASM33445v1 Actinobacteria Gordonia_soli
GCF_001083805.1_ASM108380v1 Proteobacteria Photorhabdus_luminescens
GCF_000816125.1_ASM81612v1 Proteobacteria Rhizobium_etli
GCF_000262405.1_ASM26240v1 Proteobacteria Microvirga_lotononidis
GCF_001676615.1_ASM167661v1 Proteobacteria Labrys_sp._WJW
GCF_000164885.1_ASM16488v1 Proteobacteria Ketogulonicigenium_vulgare
GCF_000790405.1_AZPAE14874 Proteobacteria Pseudomonas_aeruginosa_group
GCF_001429505.1_Root483D1 Proteobacteria Bosea_sp._Root483D1
GCF_000427885.1_ASM42788v1 Actinobacteria Glycomyces_tenuis
GCF_000426525.1_ASM42652v1 Actinobacteria Nocardioides_alkalitolerans
GCF_000760735.1_R_fas_A44a Actinobacteria Rhodococcus_fascians
0.46

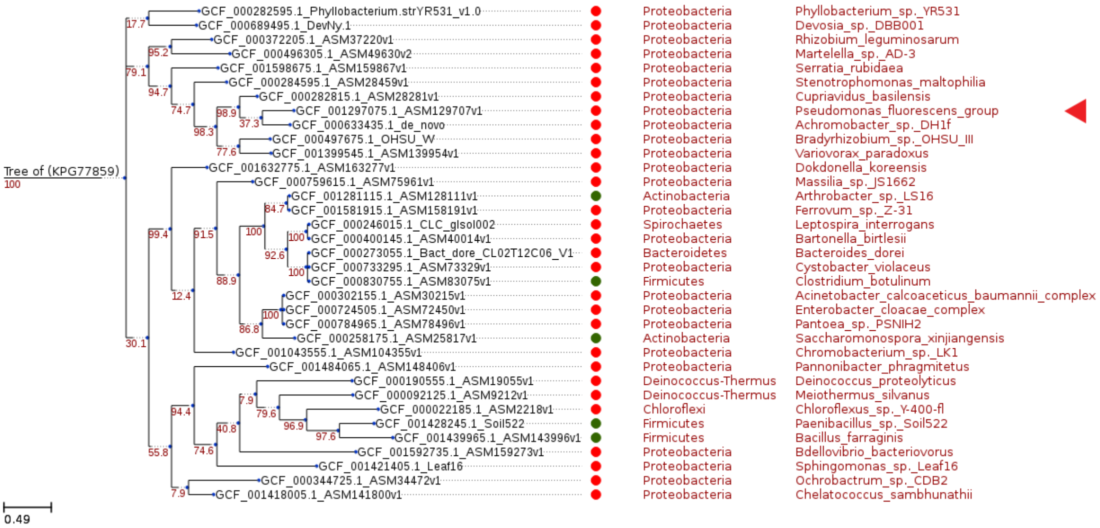
Tree of (KPG77859)
100
GCF_000282595.1_Phyllobacterium.strYR531_v1.0 Proteobacteria Phyllobacterium_sp._YR531
GCF_000689495.1_DevNy.1 Proteobacteria Devosia_sp._DBB001
GCF_000372205.1_ASM37220v1 Proteobacteria Rhizobium_leguminosarum
GCF_000496305.1_ASM49630v2 Proteobacteria Martelella_sp._AD-3
GCF_001598675.1_ASM159867v1 Proteobacteria Serratia_rubidaea
GCF_000284595.1_ASM28459v1 Proteobacteria Stenotrophomonas_maltophilia
GCF_000282815.1_ASM28281v1 Proteobacteria Cupriavidus_basilensis
GCF_001297075.1_ASM129707v1 Proteobacteria Pseudomonas_fluorescens_group
GCF_000633435.1_de_novo Proteobacteria Achromobacter_sp._DH1f
GCF_000497675.1_OHSU_W Proteobacteria Bradyrhizobium_sp._OHSU_III
GCF_001399545.1_ASM139954v1 Proteobacteria Variovorax_paradoxus
GCF_001632775.1_ASM163277v1 Proteobacteria Dokdonella_koreensis
GCF_000759615.1_ASM75961v1 Proteobacteria Massilia_sp._JS1662
GCF_001281115.1_ASM128111v1 Actinobacteria Arthrobacter_sp._LS16
GCF_001581915.1_ASM158191v1 Proteobacteria Ferrovum_sp._Z-31
GCF_000246015.1_CLC_glsol002 Spirochaetes Leptospira_interrogans
GCF_000400145.1_ASM40014v1 Proteobacteria Bartonella_birtlesii
GCF_000273055.1_Bact_dore_CL02T12C06_V1 Bacteroidetes Bacteroides_dorei
GCF_000733295.1_ASM73329v1 Proteobacteria Cystobacter_violaceus
GCF_000830755.1_ASM83075v1 Firmicutes Clostridium_botulinum
GCF_000302155.1_ASM30215v1 Proteobacteria Acinetobacter_calcoaceticus_baumannii_complex
GCF_000724505.1_ASM72450v1 Proteobacteria Enterobacter_cloacae_complex
GCF_000784965.1_ASM78496v1 Proteobacteria Pantoea_sp._PSNIH2
GCF_000258175.1_ASM25817v1 Actinobacteria Saccharomonospora_xinjiangensis
GCF_001043555.1_ASM104355v1 Proteobacteria Chromobacterium_sp._LK1
GCF_001484065.1_ASM148406v1 Proteobacteria Pannonibacter_phragmitetus
GCF_000190555.1_ASM19055v1 Deinococcus-Thermus Deinococcus_proteolyticus
GCF_000092125.1_ASM9212v1 Deinococcus-Thermus Meiothermus_silvanus
GCF_000022185.1_ASM2218v1 Chloroflexi Chloroflexus_sp._Y-400-fl
GCF_001428245.1_Soil522 Firmicutes Paenibacillus_sp._Soil522
GCF_001439965.1_ASM143996v1 Firmicutes Bacillus_farraginis
GCF_001592735.1_ASM159273v1 Proteobacteria Bdellovibrio_bacteriovorus
GCF_001421405.1_Leaf16 Proteobacteria Sphingomonas_sp._Leaf16
GCF_000344725.1_ASM34472v1 Proteobacteria Ochrobactrum_sp._CDB2
GCF_001418005.1_ASM141800v1 Proteobacteria Chelatococcus_sambhunathii
0.49

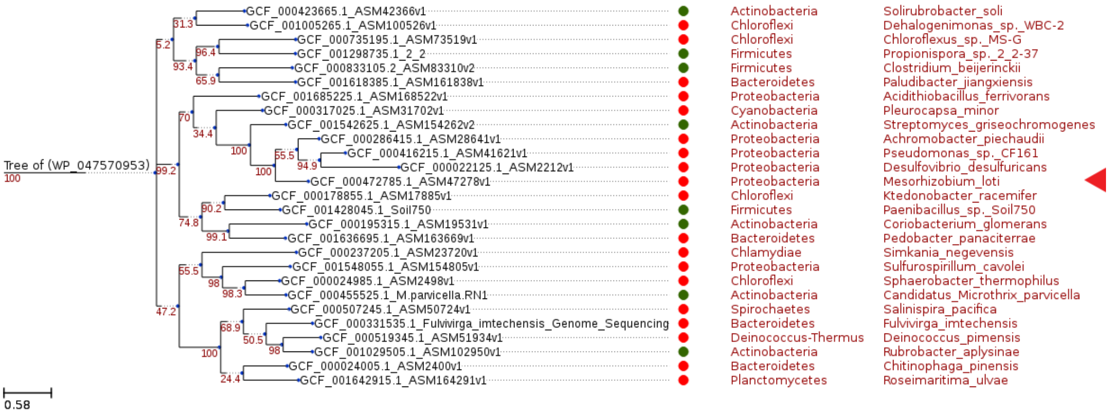
Tree of (WP_047570953)
100
GCF_000423665.1_ASM42366v1 Actinobacteria Solirubrobacter_soli
GCF_001005265.1_ASM100526v1 Chloroflexi Dehalogenimonas_sp._WBC-2
GCF_000735195.1_ASM73519v1 Chloroflexi Chloroflexus_sp._MS-G
GCF_001298735.1_2_2 Firmicutes Propionispora_sp._2_2-37
GCF_000833105.2_ASM83310v2 Firmicutes Clostridium_beijerinckii
GCF_001618385.1_ASM161838v1 Bacteroidetes Paludibacter_jiangxiensis
GCF_001685225.1_ASM168522v1 Proteobacteria Acidithiobacillus_ferrivorans
GCF_000317025.1_ASM31702v1 Cyanobacteria Pleurocapsa_minor
GCF_001542625.1_ASM154262v2 Actinobacteria Streptomyces_griseochromogenes
GCF_000286415.1_ASM28641v1 Proteobacteria Achromobacter_piechaudii
GCF_000416215.1_ASM41621v1 Proteobacteria Pseudomonas_sp._CF161
GCF_000022125.1_ASM2212v1 Proteobacteria Desulfovibrio_desulfuricans
GCF_000472785.1_ASM47278v1 Proteobacteria Mesorhizobium_loti
GCF_000178855.1_ASM17885v1 Chloroflexi Ktedonobacter_racemifer
GCF_001428045.1_Soil750 Firmicutes Paenibacillus_sp._Soil750
GCF_000195315.1_ASM19531v1 Actinobacteria Coriobacterium_glomerans
GCF_001636695.1_ASM163669v1 Bacteroidetes Pedobacter_panaciterrae
GCF_000237205.1_ASM23720v1 Chlamydiae Simkania_negevensis
GCF_001548055.1_ASM154805v1 Proteobacteria Sulfurospirillum_cavolei
GCF_000024985.1_ASM2498v1 Chloroflexi Sphaerobacter_thermophilus
GCF_000455525.1_M.parvicella.RN1 Actinobacteria Candidatus_Microthrix_parvicella
GCF_000507245.1_ASM50724v1 Spirochaetes Salinispira_pacifica
GCF_000331535.1_Fulvivirga_imtechensis_Genome_Sequencing Bacteroidetes Fulvivirga_imtechensis
GCF_000519345.1_ASM51934v1 Deinococcus-Thermus Deinococcus_pimensis
GCF_001029505.1_ASM102950v1 Actinobacteria Rubrobacter_aplysinae
GCF_000024005.1_ASM2400v1 Bacteroidetes Chitinophaga_pinensis
GCF_001642915.1_ASM164291v1 Planctomycetes Roseimaritima_ulvae
0.58

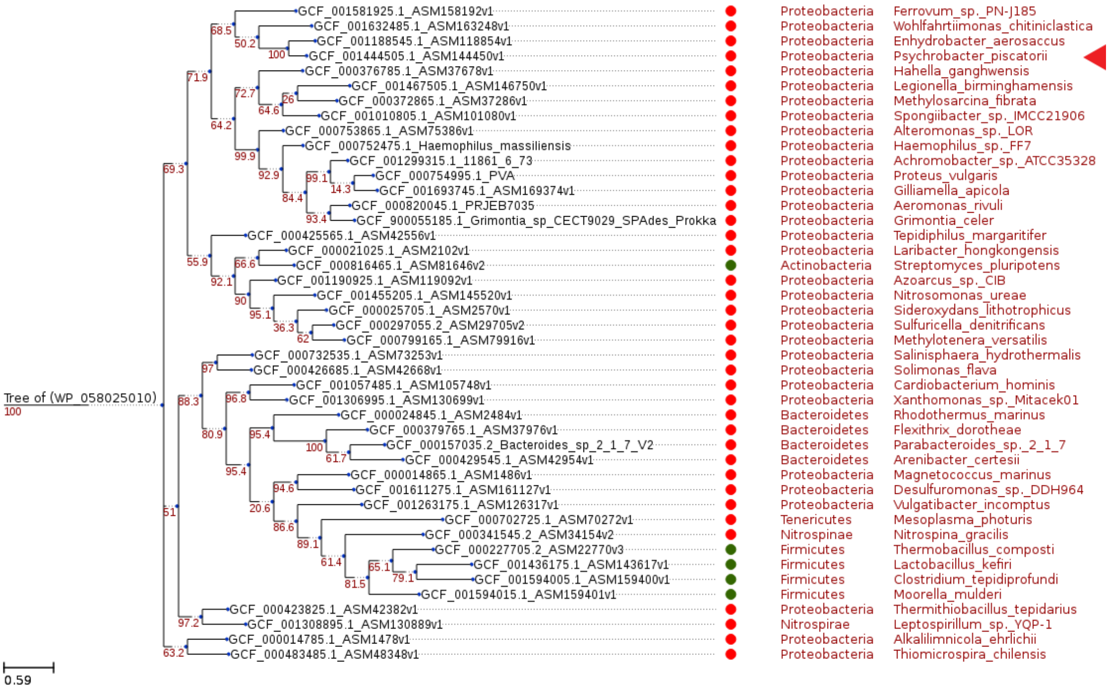
Tree of (WP_058025010)
100
0.59
GCF_001581925.1_ASM158192v1 Proteobacteria Ferrovum_sp._PN-J185
GCF_001632485.1_ASM163248v1 Proteobacteria Wohlfahrtiimonas_chitiniclastica
GCF_001188545.1_ASM118854v1 Proteobacteria Enhydrobacter_aerosaccus
GCF_001444505.1_ASM144450v1 Proteobacteria Psychrobacter_piscatorii
GCF_000376785.1_ASM37678v1 Proteobacteria Hahella_ganghwensis
GCF_001467505.1_ASM146750v1 Proteobacteria Legionella_birminghamensis
GCF_000372865.1_ASM37286v1 Proteobacteria Methylosarcina_fibrata
GCF_001010805.1_ASM101080v1 Proteobacteria Spongiibacter_sp._IMCC21906
GCF_000753865.1_ASM75386v1 Proteobacteria Alteromonas_sp._LOR
GCF_000752475.1_Haemophilus_massiliensis Proteobacteria Haemophilus_sp._FF7
GCF_001299315.1_11861_6_73 Proteobacteria Achromobacter_sp._ATCC35328
GCF_000754995.1_PVA Proteobacteria Proteus_vulgaris
GCF_001693745.1_ASM169374v1 Proteobacteria Gilliamella_apicola
GCF_000820045.1_PRJEB7035 Proteobacteria Aeromonas_rivuli
GCF_900055185.1_Grimontia_sp_CECT9029_SPAdes_Prokka Proteobacteria Grimontia_celer
GCF_000425565.1_ASM42556v1 Proteobacteria Tepidiphilus_margaritifer
GCF_000021025.1_ASM2102v1 Proteobacteria Laribacter_hongkongensis
GCF_000816465.1_ASM81646v2 Actinobacteria Streptomyces_pluripotens
GCF_001190925.1_ASM119092v1 Proteobacteria Azoarcus_sp._CIB
GCF_001455205.1_ASM145520v1 Proteobacteria Nitrosomonas_ureae
GCF_000025705.1_ASM2570v1 Proteobacteria Sideroxydans_lithotrophicus
GCF_000297055.2_ASM29705v2 Proteobacteria Sulfuricella_denitrificans
GCF_000799165.1_ASM79916v1 Proteobacteria Methylotenera_versatilis
GCF_000732535.1_ASM73253v1 Proteobacteria Salinisphaera_hydrothermalis
GCF_000426685.1_ASM42668v1 Proteobacteria Solimonas_flava
GCF_001057485.1_ASM105748v1 Proteobacteria Cardiobacterium_hominis
GCF_001306995.1_ASM130699v1 Proteobacteria Xanthomonas_sp._Mitacek01
GCF_000024845.1_ASM2484v1 Bacteroidetes Rhodothermus_marinus
GCF_000379765.1_ASM37976v1 Bacteroidetes Flexithrix_dorotheae
GCF_000157035.2_Bacteroides_sp_2_1_7_V2 Bacteroidetes Parabacteroides_sp._2_1_7
GCF_000429545.1_ASM42954v1 Bacteroidetes Arenibacter_certesii
GCF_000014865.1_ASM1486v1 Proteobacteria Magnetococcus_marinus
GCF_001611275.1_ASM161127v1 Proteobacteria Desulfuromonas_sp._DDH964
GCF_001263175.1_ASM126317v1 Proteobacteria Vulgatibacter_incomptus
GCF_000702725.1_ASM70272v1 Tenericutes Mesoplasma_photuris
GCF_000341545.2_ASM34154v2 Nitrospinae Nitrospina_gracilis
GCF_000227705.2_ASM22770v3 Firmicutes Thermobacillus_composti
GCF_001436175.1_ASM143617v1 Firmicutes Lactobacillus_kefiri
GCF_001594005.1_ASM159400v1 Firmicutes Clostridium_tepidiprofundi
GCF_001594015.1_ASM159401v1 Firmicutes Moorella_mulderi
GCF_000423825.1_ASM42382v1 Proteobacteria Thermithiobacillus_tepidarius
GCF_001308895.1_ASM130889v1 Nitrospirae Leptospirillum_sp._YQP-1
GCF_000014785.1_ASM1478v1 Proteobacteria Alkalilimnicola_ehrlichii
GCF_000483485.1_ASM48348v1 Proteobacteria Thiomicrospira_chilensis

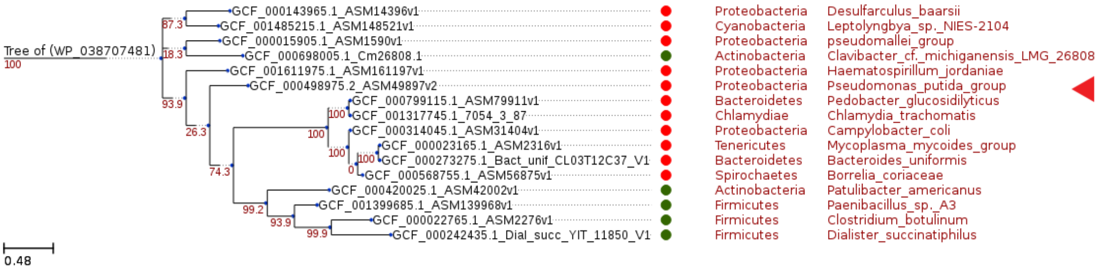
Tree of (WP_038707481)
100
0.48
GCF_000143965.1_ASM14396v1 Proteobacteria Desulfarculus_baarsii
GCF_001485215.1_ASM148521v1 Cyanobacteria Leptolyngbya_sp._NIES-2104
GCF_000015905.1_ASM1590v1 Proteobacteria pseudomallei_group
GCF_000698005.1_Cm26808.1 Actinobacteria Clavibacter_cf._michiganensis_LMG_26808
GCF_001611975.1_ASM161197v1 Proteobacteria Haematospirillum_jordaniae
GCF_000498975.2_ASM49897v2 Proteobacteria Pseudomonas_putida_group
GCF_000799115.1_ASM79911v1 Bacteroidetes Pedobacter_glucosidilyticus
GCF_001317745.1_7054_3_87 Chlamydiae Chlamydia_trachomatis
GCF_000314045.1_ASM31404v1 Proteobacteria Campylobacter_coli
GCF_000023165.1_ASM2316v1 Tenericutes Mycoplasma_mycoides_group
GCF_000273275.1_Bact_unif_CL03T12C37_V1 Bacteroidetes Bacteroides_uniformis
GCF_000568755.1_ASM56875v1 Spirochaetes Borrelia_coriaceae
GCF_000420025.1_ASM42002v1 Actinobacteria Patulibacter_americanus
GCF_001399685.1_ASM139968v1 Firmicutes Paenibacillus_sp._A3
GCF_000022765.1_ASM2276v1 Firmicutes Clostridium_botulinum
GCF_000242435.1_Dial_succ_YIT_11850_V1 Firmicutes Dialister_succinatiphilus

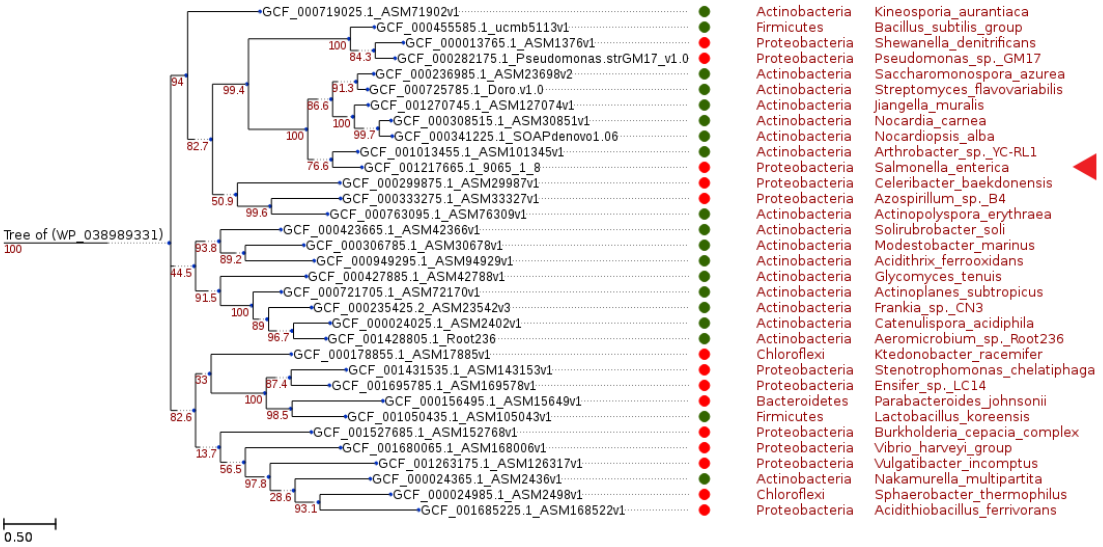
Tree of (WP_038989331)
100
0.50
GCF_000719025.1_ASM71902v1 Actinobacteria Kineosporia_aurantiaca
GCF_000455585.1_ucmb5113v1 Firmicutes Bacillus_subtilis_group
GCF_000013765.1_ASM1376v1 Proteobacteria Shewanella_denitrificans
GCF_000282175.1_Pseudomonas.strGM17_v1.0 Proteobacteria Pseudomonas_sp._GM17
GCF_000236985.1_ASM23698v2 Actinobacteria Saccharomonospora_azurea
GCF_000725785.1_Doro.v1.0 Actinobacteria Streptomyces_flavovariabilis
GCF_001270745.1_ASM127074v1 Actinobacteria Jiangella_muralis
GCF_000308515.1_ASM30851v1 Actinobacteria Nocardia_carnea
GCF_000341225.1_SOAPdenovo1.06 Actinobacteria Nocardiopsis_alba
GCF_001013455.1_ASM101345v1 Actinobacteria Arthrobacter_sp._YC-RL1
GCF_001217665.1_9065_1_8 Proteobacteria Salmonella_enterica
GCF_000299875.1_ASM29987v1 Proteobacteria Celeribacter_baekdonensis
GCF_000333275.1_ASM33327v1 Proteobacteria Azospirillum_sp._B4
GCF_000763095.1_ASM76309v1 Actinobacteria Actinopolyspora_erythraea
GCF_000423665.1_ASM42366v1 Actinobacteria Solirubrobacter_soli
GCF_000306785.1_ASM30678v1 Actinobacteria Modestobacter_marinus
GCF_000949295.1_ASM94929v1 Actinobacteria Acidithrix_ferrooxidans
GCF_000427885.1_ASM42788v1 Actinobacteria Glycomyces_tenuis
GCF_000721705.1_ASM72170v1 Actinobacteria Actinoplanes_subtropicus
GCF_000235425.2_ASM23542v3 Actinobacteria Frankia_sp._CN3
GCF_000024025.1_ASM2402v1 Actinobacteria Catenulispora_acidiphila
GCF_001428805.1_Root236 Actinobacteria Aeromicrobium_sp._Root236
GCF_000178855.1_ASM17885v1 Chloroflexi Ktedonobacter_racemifer
GCF_001431535.1_ASM143153v1 Proteobacteria Stenotrophomonas_chelatiphaga
GCF_001695785.1_ASM169578v1 Proteobacteria Ensifer_sp._LC14
GCF_000156495.1_ASM15649v1 Bacteroidetes Parabacteroides_johnsonii
GCF_001050435.1_ASM105043v1 Firmicutes Lactobacillus_koreensis
GCF_001527685.1_ASM152768v1 Proteobacteria Burkholderia_cepacia_complex
GCF_001680065.1_ASM168006v1 Proteobacteria Vibrio_harveyi_group
GCF_001263175.1_ASM126317v1 Proteobacteria Vulgatibacter_incomptus
GCF_000024365.1_ASM2436v1 Actinobacteria Nakamurella_multipartita
GCF_000024985.1_ASM2498v1 Chloroflexi Sphaerobacter_thermophilus
GCF_001685225.1_ASM168522v1 Proteobacteria Acidithiobacillus_ferrivorans

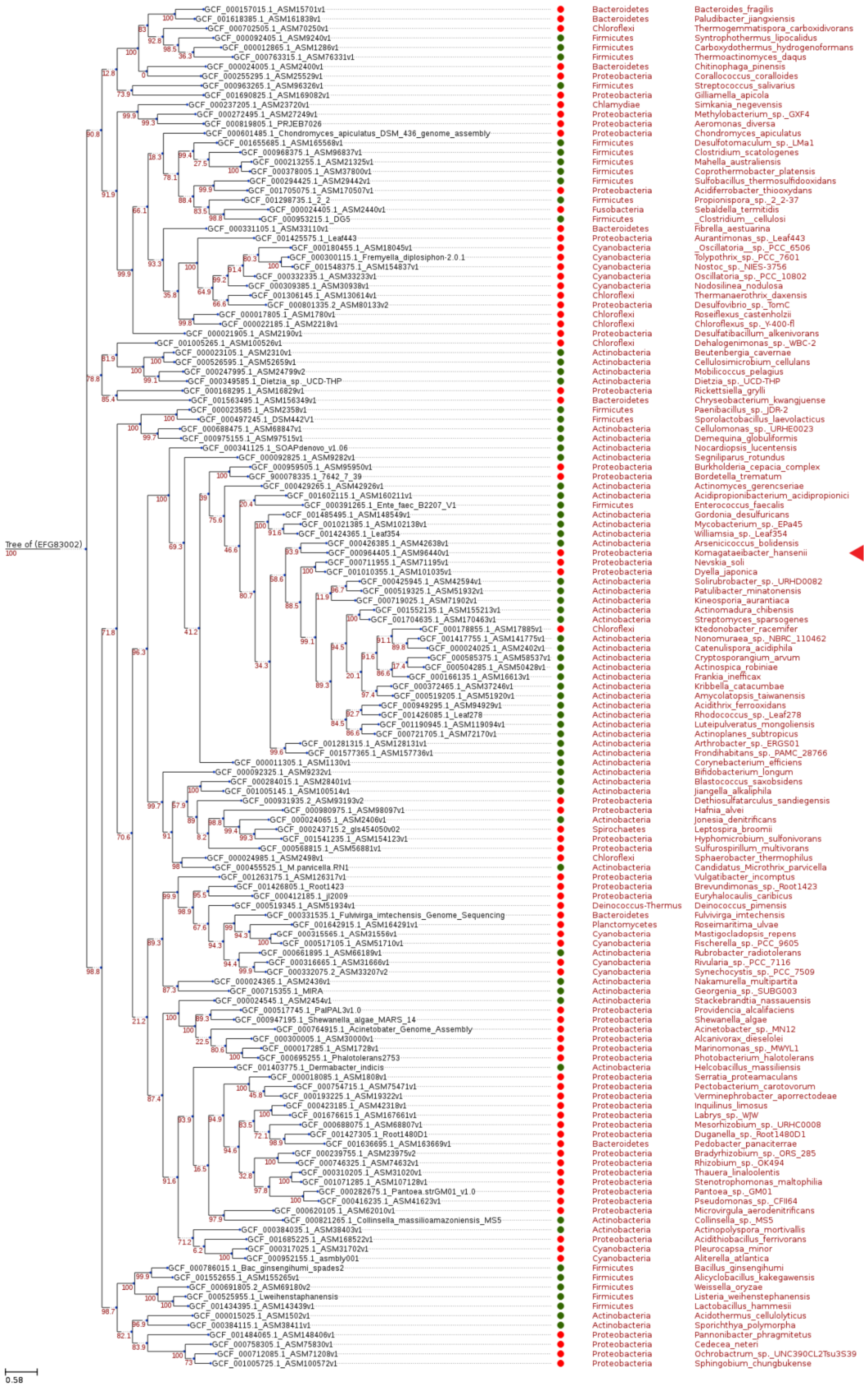

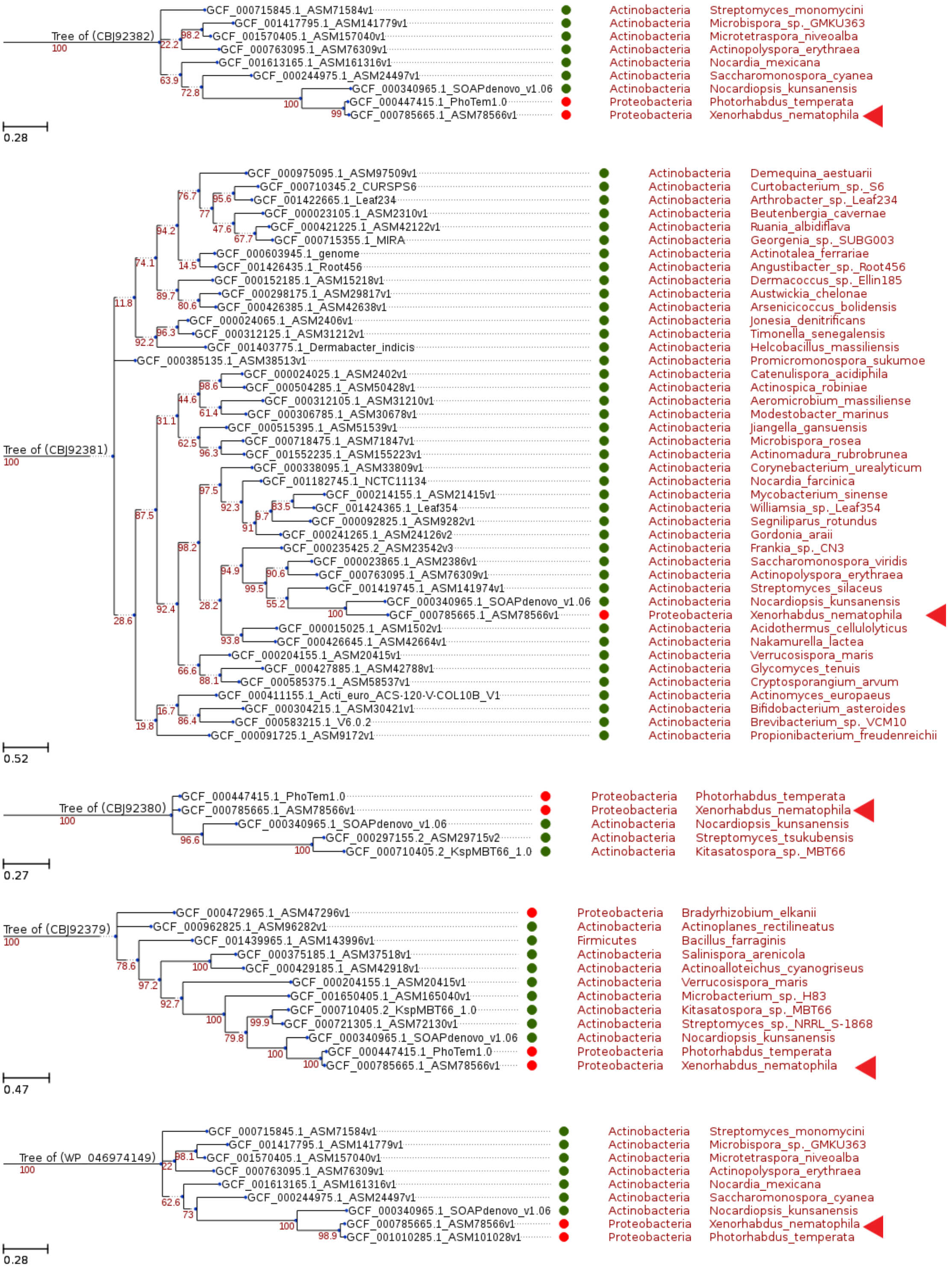
Tree of (CBJ92382)
GCF_000715845.1_ASM71584v1 Actinobacteria Streptomyces_monomycini
GCF_001417795.1_ASM141779v1 Actinobacteria Microbispora_sp._GMKU363
GCF_001570405.1_ASM157040v1 Actinobacteria Microtetraspora_niveoalba
GCF_000763095.1_ASM76309v1 Actinobacteria Actinopolyspora_erythraea
GCF_001613165.1_ASM161316v1 Actinobacteria Nocardia_mexicana
GCF_000244975.1_ASM24497v1 Actinobacteria Saccharomonospora_cyanea
GCF_000340965.1_SOAPdenovo_v1.06 Actinobacteria Nocardiopsis_kunsanensis
GCF_000447415.1_PhoTem1.0 Proteobacteria Photorhabdus_temperata
GCF_000785665.1_ASM78566v1 Proteobacteria Xenorhabdus_nematophila
0.28
Tree of (CBJ92381)
GCF_000975095.1_ASM97509v1 Actinobacteria Demequina_aestuarii
GCF_000710345.2_CURSPS6 Actinobacteria Curtobacterium_sp._S6
GCF_001422665.1_Leaf234 Actinobacteria Arthrobacter_sp._Leaf234
GCF_000023105.1_ASM2310v1 Actinobacteria Beutenbergia_cavernae
GCF_000421225.1_ASM42122v1 Actinobacteria Ruania_albidiflava
GCF_000715355.1_MIRA Actinobacteria Georgenia_sp._SUBG003
GCF_000603945.1_genome Actinobacteria Actinotalea_ferrariae
GCF_001426435.1_Root456 Actinobacteria Angustibacter_sp._Root456
GCF_000152185.1_ASM15218v1 Actinobacteria Dermacoccus_sp._Ellin185
GCF_000298175.1_ASM29817v1 Actinobacteria Austwickia_chelonae
GCF_000426385.1_ASM42638v1 Actinobacteria Arsenicicoccus_bolidensis
GCF_000024065.1_ASM2406v1 Actinobacteria Jonesia_denitrificans
GCF_000312125.1_ASM31212v1 Actinobacteria Timonella_senegalensis
GCF_001403775.1_Dermabacter_indicis Actinobacteria Helcobacillus_massiliensis
GCF_000385135.1_ASM38513v1 Actinobacteria Promicromonospora_sukumoe
GCF_000024025.1_ASM2402v1 Actinobacteria Catenulispora_acidiphila
GCF_000504285.1_ASM50428v1 Actinobacteria Actinospica_robiniae
GCF_000312105.1_ASM31210v1 Actinobacteria Aeromicrobium_massiliense
GCF_000306785.1_ASM30678v1 Actinobacteria Modestobacter_marinus
GCF_000515395.1_ASM51539v1 Actinobacteria Jiangella_gansuensis
GCF_000718475.1_ASM71847v1 Actinobacteria Microbispora_rosea
GCF_001552235.1_ASM155223v1 Actinobacteria Actinomadura_rubrobrunea
GCF_000338095.1_ASM33809v1 Actinobacteria Corynebacterium_urealyticum
GCF_001182745.1_NCTC11134 Actinobacteria Nocardia_farcinica
GCF_000214155.1_ASM21415v1 Actinobacteria Mycobacterium_sinense
GCF_001424365.1_Leaf354 Actinobacteria Williamsia_sp._Leaf354
GCF_000092825.1_ASM9282v1 Actinobacteria Segniliparus_rotundus
GCF_000241265.1_ASM24126v2 Actinobacteria Gordonia_araii
GCF_000235425.2_ASM23542v3 Actinobacteria Frankia_sp._CN3
GCF_000023865.1_ASM2386v1 Actinobacteria Saccharomonospora_viridis
GCF_000763095.1_ASM76309v1 Actinobacteria Actinopolyspora_erythraea
GCF_001419745.1_ASM141974v1 Actinobacteria Streptomyces_silaceus
GCF_000340965.1_SOAPdenovo_v1.06 Actinobacteria Nocardiopsis_kunsanensis
GCF_000785665.1_ASM78566v1 Proteobacteria Xenorhabdus_nematophila
GCF_000015025.1_ASM1502v1 Actinobacteria Acidothermus_cellulolyticus
GCF_000426645.1_ASM42664v1 Actinobacteria Nakamurella_lactea
GCF_000204155.1_ASM20415v1 Actinobacteria Verrucosispora_maris
GCF_000427885.1_ASM42788v1 Actinobacteria Glycomyces_tenuis
GCF_000585375.1_ASM58537v1 Actinobacteria Cryptosporangium_arvum
GCF_000411155.1_Acti_euro_ACS-120-V-COL10B_V1 Actinobacteria Actinomyces_europaeus
GCF_000304215.1_ASM30421v1 Actinobacteria Bifidobacterium_asteroides
GCF_000583215.1_V6.0.2 Actinobacteria Brevibacterium_sp._VCM10
GCF_000091725.1_ASM9172v1 Actinobacteria Propionibacterium_freudenreichii
0.52
Tree of (CBJ92380)
GCF_000447415.1_PhoTem1.0 Proteobacteria Photorhabdus_temperata
GCF_000785665.1_ASM78566v1 Proteobacteria Xenorhabdus_nematophila
GCF_000340965.1_SOAPdenovo_v1.06 Actinobacteria Nocardiopsis_kunsanensis
GCF_000297155.2_ASM29715v2 Actinobacteria Streptomyces_tsukubensis
GCF_000710405.2_KspMBT66_1.0 Actinobacteria Kitasatospora_sp._MBT66
0.27
Tree of (CBJ92379)
GCF_000472965.1_ASM47296v1 Proteobacteria Bradyrhizobium_elkanii
GCF_000962825.1_ASM96282v1 Actinobacteria Actinoplanes_rectilineatus
GCF_001439965.1_ASM143996v1 Firmicutes Bacillus_farraginis
GCF_000375185.1_ASM37518v1 Actinobacteria Salinispora_arenicola
GCF_000429185.1_ASM42918v1 Actinobacteria Actinoalloteichus_cyanogriseus
GCF_000204155.1_ASM20415v1 Actinobacteria Verrucosispora_maris
GCF_001650405.1_ASM165040v1 Actinobacteria Microbacterium_sp._H83
GCF_000710405.2_KspMBT66_1.0 Actinobacteria Kitasatospora_sp._MBT66
GCF_000721305.1_ASM72130v1 Actinobacteria Streptomyces_sp._NRRL_S-1868
GCF_000340965.1_SOAPdenovo_v1.06 Actinobacteria Nocardiopsis_kunsanensis
GCF_000447415.1_PhoTem1.0 Proteobacteria Photorhabdus_temperata
GCF_000785665.1_ASM78566v1 Proteobacteria Xenorhabdus_nematophila
0.47
Tree of (WP_046974149)
GCF_000715845.1_ASM71584v1 Actinobacteria Streptomyces_monomycini
GCF_001417795.1_ASM141779v1 Actinobacteria Microbispora_sp._GMKU363
GCF_001570405.1_ASM157040v1 Actinobacteria Microtetraspora_niveoalba
GCF_000763095.1_ASM76309v1 Actinobacteria Actinopolyspora_erythraea
GCF_001613165.1_ASM161316v1 Actinobacteria Nocardia_mexicana
GCF_000244975.1_ASM24497v1 Actinobacteria Saccharomonospora_cyanea
GCF_000340965.1_SOAPdenovo_v1.06 Actinobacteria Nocardiopsis_kunsanensis
GCF_000785665.1_ASM78566v1 Proteobacteria Xenorhabdus_nematophila
GCF_001010285.1_ASM101028v1 Proteobacteria Photorhabdus_temperata
0.28

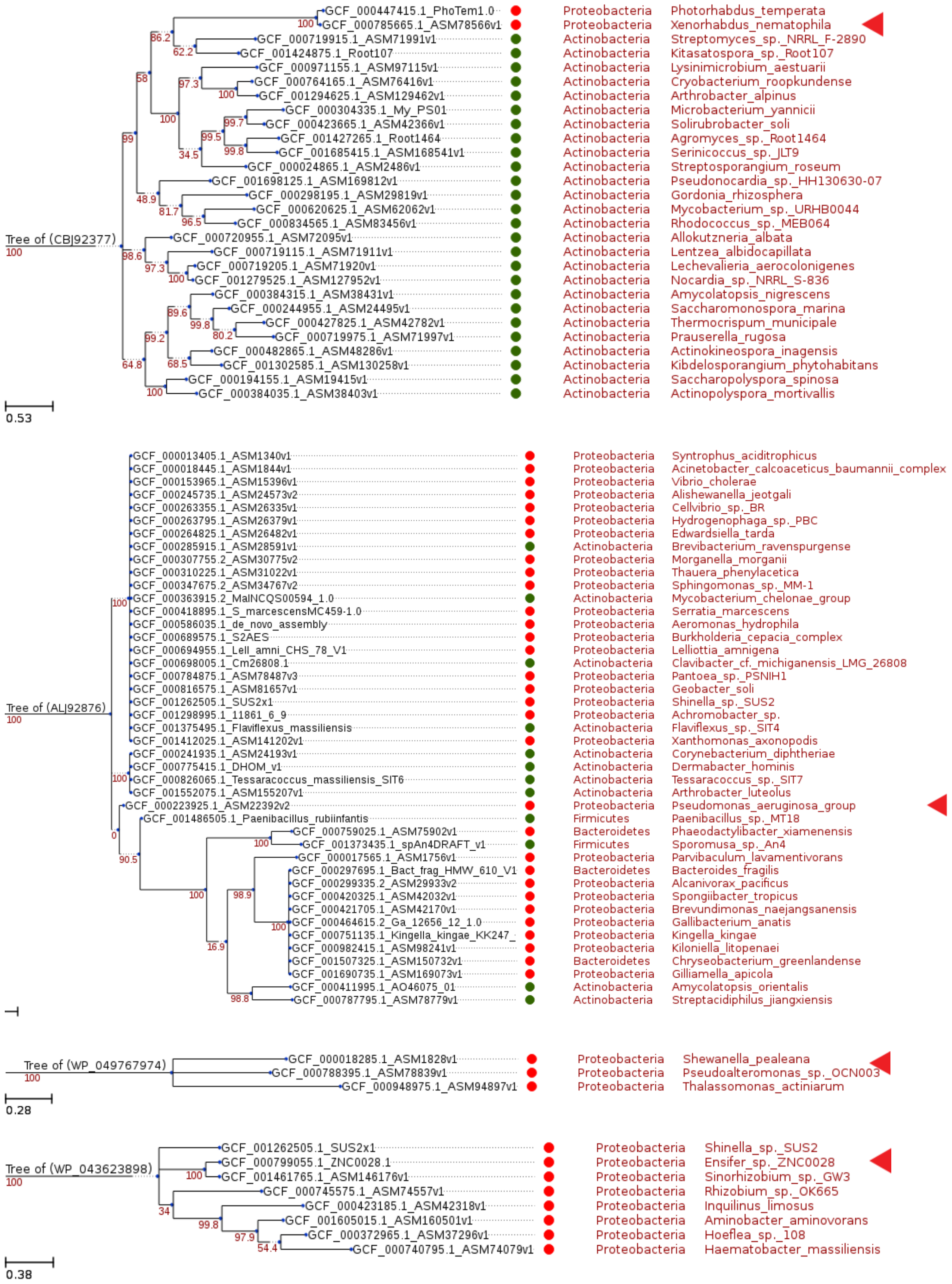
Tree of (CBJ92377)
100
GCF_000447415.1_PhoTem1.0 Proteobacteria Photorhabdus_temperata
GCF_000785665.1_ASM78566v1 Proteobacteria Xenorhabdus_nematophila
GCF_000719915.1_ASM71991v1 Actinobacteria Streptomyces_sp._NRRL_F-2890
GCF_001424875.1_Root107 Actinobacteria Kitasatospora_sp._Root107
GCF_000971155.1_ASM97115v1 Actinobacteria Lysinimicrobium_aestuarii
GCF_000764165.1_ASM76416v1 Actinobacteria Cryobacterium_roopkundense
GCF_001294625.1_ASM129462v1 Actinobacteria Arthrobacter_alpinus
GCF_000304335.1_My_PS01 Actinobacteria Microbacterium_yannicii
GCF_000423665.1_ASM42366v1 Actinobacteria Solirubrobacter_soli
GCF_001427265.1_Root1464 Actinobacteria Agromyces_sp._Root1464
GCF_001685415.1_ASM168541v1 Actinobacteria Serinicoccus_sp._JLT9
GCF_000024865.1_ASM2486v1 Actinobacteria Streptosporangium_roseum
GCF_001698125.1_ASM169812v1 Actinobacteria Pseudonocardia_sp._HH130630-07
GCF_000298195.1_ASM29819v1 Actinobacteria Gordonia_rhizosphera
GCF_000620625.1_ASM62062v1 Actinobacteria Mycobacterium_sp._URHB0044
GCF_000834565.1_ASM83456v1 Actinobacteria Rhodococcus_sp._MEB064
GCF_000720955.1_ASM72095v1 Actinobacteria Allokutzneria_albata
GCF_000719115.1_ASM71911v1 Actinobacteria Lentzea_albidocapillata
GCF_000719205.1_ASM71920v1 Actinobacteria Lechevalieria_aerocolonigenes
GCF_001279525.1_ASM127952v1 Actinobacteria Nocardia_sp._NRRL_S-836
GCF_000384315.1_ASM38431v1 Actinobacteria Amycolatopsis_nigrescens
GCF_000244955.1_ASM24495v1 Actinobacteria Saccharomonospora_marina
GCF_000427825.1_ASM42782v1 Actinobacteria Thermocrispum_municipale
GCF_000719975.1_ASM71997v1 Actinobacteria Prauserella_rugosa
GCF_000482865.1_ASM48286v1 Actinobacteria Actinokineospora_inagensis
GCF_001302585.1_ASM130258v1 Actinobacteria Kibdelosporangium_phytohabitans
GCF_000194155.1_ASM19415v1 Actinobacteria Saccharopolyspora_spinosa
GCF_000384035.1_ASM38403v1 Actinobacteria Actinopolyspora_mortivallis
0.53
Tree of (ALJ92876)
100
GCF_000013405.1_ASM1340v1 Proteobacteria Syntrophus_aciditrophicus
GCF_000018445.1_ASM1844v1 Proteobacteria Acinetobacter_calcoaceticus_baumannii_complex
GCF_000153965.1_ASM15396v1 Proteobacteria Vibrio_cholerae
GCF_000245735.1_ASM24573v2 Proteobacteria Alishewanella_jeotgali
GCF_000263355.1_ASM26335v1 Proteobacteria Cellvibrio_sp._BR
GCF_000263795.1_ASM26379v1 Proteobacteria Hydrogenophaga_sp._PBC
GCF_000264825.1_ASM26482v1 Proteobacteria Edwardsiella_tarda
GCF_000285915.1_ASM28591v1 Actinobacteria Brevibacterium_ravenspurgense
GCF_000307755.2_ASM30775v2 Proteobacteria Morganella_morganii
GCF_000310225.1_ASM31022v1 Proteobacteria Thauera_phenylacetica
GCF_000347675.2_ASM34767v2 Proteobacteria Sphingomonas_sp._MM-1
GCF_000363915.2_MalNCQS00594_1.0 Actinobacteria Mycobacterium_chelonae_group
GCF_000418895.1_S_marcescensMC459-1.0 Proteobacteria Serratia_marcescens
GCF_000586035.1_de_novo_assembly Proteobacteria Aeromonas_hydrophila
GCF_000689575.1_S2AES Proteobacteria Burkholderia_cepacia_complex
GCF_000694955.1_Lell_amni_CHS_78_V1 Proteobacteria Lelliottia_amnigena
GCF_000698005.1_Cm26808.1 Actinobacteria Clavibacter_cf._michiganensis_LMG_26808
GCF_000784875.1_ASM78487v3 Proteobacteria Pantoea_sp._PSNIH1
GCF_000816575.1_ASM81657v1 Proteobacteria Geobacter_soli
GCF_001262505.1_SUS2x1 Proteobacteria Shinella_sp._SUS2
GCF_001298995.1_11861_6_9 Proteobacteria Achromobacter_sp.
GCF_001375495.1_Flaviflexus_massiliensis Actinobacteria Flaviflexus_sp._SIT4
GCF_001412025.1_ASM141202v1 Proteobacteria Xanthomonas_axonopodis
GCF_000241935.1_ASM24193v1 Actinobacteria Corynebacterium_diphtheriae
GCF_000775415.1_DHOM_v1 Actinobacteria Dermabacter_hominis
GCF_000826065.1_Tessaracoccus_massiliensis_SIT6 Actinobacteria Tessaracoccus_sp._SIT7
GCF_001552075.1_ASM155207v1 Actinobacteria Arthrobacter_luteolus
GCF_000223925.1_ASM22392v2 Proteobacteria Pseudomonas_aeruginosa_group
GCF_001486505.1_Paenibacillus_rubiinfantis Firmicutes Paenibacillus_sp._MT18
GCF_000759025.1_ASM75902v1 Bacteroidetes Phaeodactylibacter_xiamenensis
GCF_001373435.1_spAn4DRAFT_v1 Firmicutes Sporomusa_sp._An4
GCF_000017565.1_ASM1756v1 Proteobacteria Parvibaculum_lavamentivorans
GCF_000297695.1_Bact_frag_HMW_610_V1 Bacteroidetes Bacteroides_fragilis
GCF_000299335.2_ASM29933v2 Proteobacteria Alcanivorax_pacificus
GCF_000420325.1_ASM42032v1 Proteobacteria Spongiibacter_tropicus
GCF_000421705.1_ASM42170v1 Proteobacteria Brevundimonas_naejangsanensis
GCF_000464615.2_Ga_12656_12_1.0 Proteobacteria Gallibacterium_anatis
GCF_000751135.1_Kingella_kingae_KK247_ Proteobacteria Kingella_kingae
GCF_000982415.1_ASM98241v1 Proteobacteria Kiloniella_litopenaei
GCF_001507325.1_ASM150732v1 Bacteroidetes Chryseobacterium_greenlandense
GCF_001690735.1_ASM169073v1 Proteobacteria Gilliamella_apicola
GCF_000411995.1_AO46075_01 Actinobacteria Amycolatopsis_orientalis
GCF_000787795.1_ASM78779v1 Actinobacteria Streptacidiphilus_jiangxiensis
Tree of (WP_049767974)
100
GCF_000018285.1_ASM1828v1 Proteobacteria Shewanella_pealeana
GCF_000788395.1_ASM78839v1 Proteobacteria Pseudoalteromonas_sp._OCN003
GCF_000948975.1_ASM94897v1 Proteobacteria Thalassomonas_actiniarum
0.28
Tree of (WP_043623898)
100
GCF_001262505.1_SUS2x1 Proteobacteria Shinella_sp._SUS2
GCF_000799055.1_ZNC0028.1 Proteobacteria Ensifer_sp._ZNC0028
GCF_001461765.1_ASM146176v1 Proteobacteria Sinorhizobium_sp._GW3
GCF_000745575.1_ASM74557v1 Proteobacteria Rhizobium_sp._OK665
GCF_000423185.1_ASM42318v1 Proteobacteria Inquilinus_limosus
GCF_001605015.1_ASM160501v1 Proteobacteria Aminobacter_aminovorans
GCF_000372965.1_ASM37296v1 Proteobacteria Hoeflea_sp._108
GCF_000740795.1_ASM74079v1 Proteobacteria Haematobacter_massiliensis
0.38

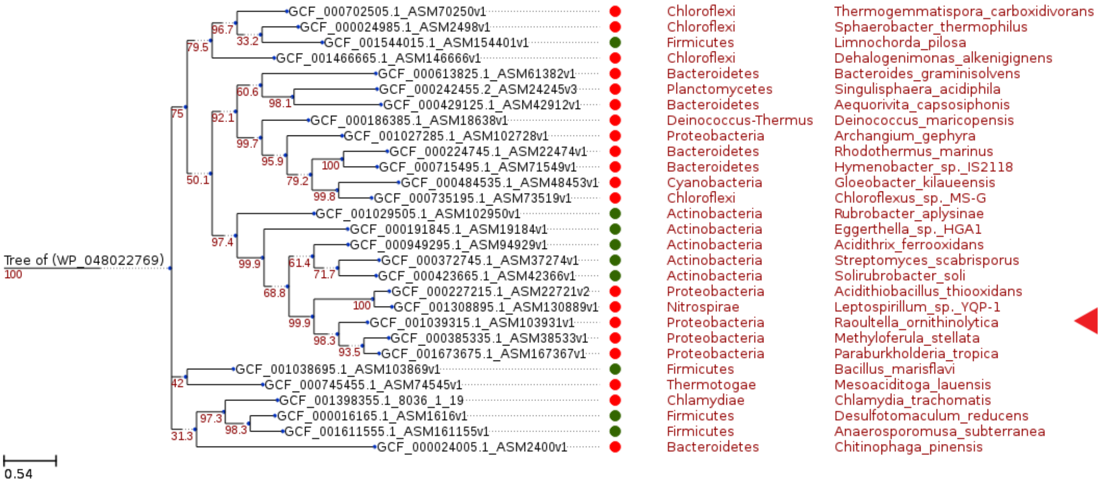
Tree of (WP_048022769)
0.54
GCF_000702505.1_ASM70250v1 Chloroflexi Thermogemmatispora_carboxidivorans
GCF_000024985.1_ASM2498v1 Chloroflexi Sphaerobacter_thermophilus
GCF_001544015.1_ASM154401v1 Firmicutes Limnochorda_pilosa
GCF_001466665.1_ASM146666v1 Chloroflexi Dehalogenimonas_alkenigignens
GCF_000613825.1_ASM61382v1 Bacteroidetes Bacteroides_graminisolvens
GCF_000242455.2_ASM24245v3 Planctomycetes Singulisphaera_acidiphila
GCF_000429125.1_ASM42912v1 Bacteroidetes Aequorivita_capsosiphonis
GCF_000186385.1_ASM18638v1 Deinococcus-Thermus Deinococcus_maricopensis
GCF_001027285.1_ASM102728v1 Proteobacteria Archangium_gephyra
GCF_000224745.1_ASM22474v1 Bacteroidetes Rhodothermus_marinus
GCF_000715495.1_ASM71549v1 Bacteroidetes Hymenobacter_sp._IS2118
GCF_000484535.1_ASM48453v1 Cyanobacteria Gloeobacter_kilaueensis
GCF_000735195.1_ASM73519v1 Chloroflexi Chloroflexus_sp._MS-G
GCF_001029505.1_ASM102950v1 Actinobacteria Rubrobacter_aplysinae
GCF_000191845.1_ASM19184v1 Actinobacteria Eggerthella_sp._HGA1
GCF_000949295.1_ASM94929v1 Actinobacteria Acidithrix_ferrooxidans
GCF_000372745.1_ASM37274v1 Actinobacteria Streptomyces_scabrisporus
GCF_000423665.1_ASM42366v1 Actinobacteria Solirubrobacter_soli
GCF_000227215.1_ASM22721v2 Proteobacteria Acidithiobacillus_thiooxidans
GCF_001308895.1_ASM130889v1 Nitrospirae Leptospirillum_sp._YQP-1
GCF_001039315.1_ASM103931v1 Proteobacteria Raoultella_ornithinolytica
GCF_000385335.1_ASM38533v1 Proteobacteria Methyloferula_stellata
GCF_001673675.1_ASM167367v1 Proteobacteria Paraburkholderia_tropica
GCF_001038695.1_ASM103869v1 Firmicutes Bacillus_marisflavi
GCF_000745455.1_ASM74545v1 Thermotogae Mesoaciditoga_lauensis
GCF_001398355.1_8036_1_19 Chlamydiae Chlamydia_trachomatis
GCF_000016165.1_ASM1616v1 Firmicutes Desulfotomaculum_reducens
GCF_001611555.1_ASM161155v1 Firmicutes Anaerosporomusa_subterranea
GCF_000024005.1_ASM2400v1 Bacteroidetes Chitinophaga_pinensis

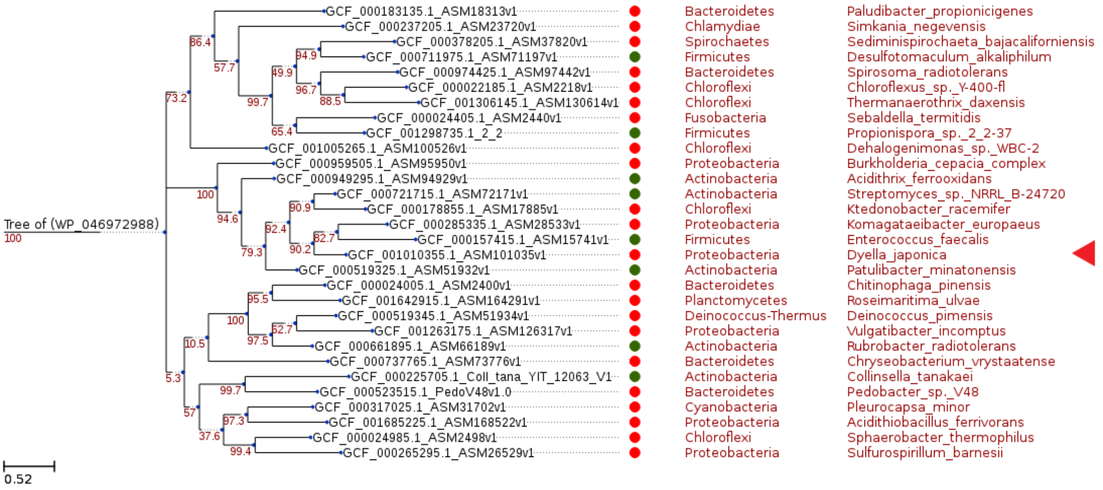
Tree of (WP_046972988)
0.52
GCF_000183135.1_ASM18313v1 Bacteroidetes Paludibacter_propionicigenes
GCF_000237205.1_ASM23720v1 Chlamydiae Simkania_negevensis
GCF_000378205.1_ASM37820v1 Spirochaetes Sediminispirochaeta_bajacaliforniensis
GCF_000711975.1_ASM71197v1 Firmicutes Desulfotomaculum_alkaliphilum
GCF_000974425.1_ASM97442v1 Bacteroidetes Spirosoma_radiotolerans
GCF_000022185.1_ASM2218v1 Chloroflexi Chloroflexus_sp._Y-400-fl
GCF_001306145.1_ASM130614v1 Chloroflexi Thermanaerothrix_daxensis
GCF_000024405.1_ASM2440v1 Fusobacteria Sebaldella_termitidis
GCF_001298735.1_2_2 Firmicutes Propionispora_sp._2_2-37
GCF_001005265.1_ASM100526v1 Chloroflexi Dehalogenimonas_sp._WBC-2
GCF_000959505.1_ASM95950v1 Proteobacteria Burkholderia_cepacia_complex
GCF_000949295.1_ASM94929v1 Actinobacteria Acidithrix_ferrooxidans
GCF_000721715.1_ASM72171v1 Actinobacteria Streptomyces_sp._NRRL_B-24720
GCF_000178855.1_ASM17885v1 Chloroflexi Ktedonobacter_racemifer
GCF_000285335.1_ASM28533v1 Proteobacteria Komagataeibacter_europaeus
GCF_000157415.1_ASM15741v1 Firmicutes Enterococcus_faecalis
GCF_001010355.1_ASM101035v1 Proteobacteria Dyella_japonica
GCF_000519325.1_ASM51932v1 Actinobacteria Patulibacter_minatonensis
GCF_000024005.1_ASM2400v1 Bacteroidetes Chitinophaga_pinensis
GCF_001642915.1_ASM164291v1 Planctomycetes Roseimaritima_ulvae
GCF_000519345.1_ASM51934v1 Deinococcus-Thermus Deinococcus_pimensis
GCF_001263175.1_ASM126317v1 Proteobacteria Vulgatibacter_incomptus
GCF_000661895.1_ASM66189v1 Actinobacteria Rubrobacter_radiotolerans
GCF_000737765.1_ASM73776v1 Bacteroidetes Chryseobacterium_vrystaatense
GCF_000225705.1_Coll_tana_YIT_12063_V1 Actinobacteria Collinsella_tanakaei
GCF_000523515.1_PedoV48v1.0 Bacteroidetes Pedobacter_sp._V48
GCF_000317025.1_ASM31702v1 Cyanobacteria Pleurocapsa_minor
GCF_001685225.1_ASM168522v1 Proteobacteria Acidithiobacillus_ferrivorans
GCF_000024985.1_ASM2498v1 Chloroflexi Sphaerobacter_thermophilus
GCF_000265295.1_ASM26529v1 Proteobacteria Sulfurospirillum_barnesii

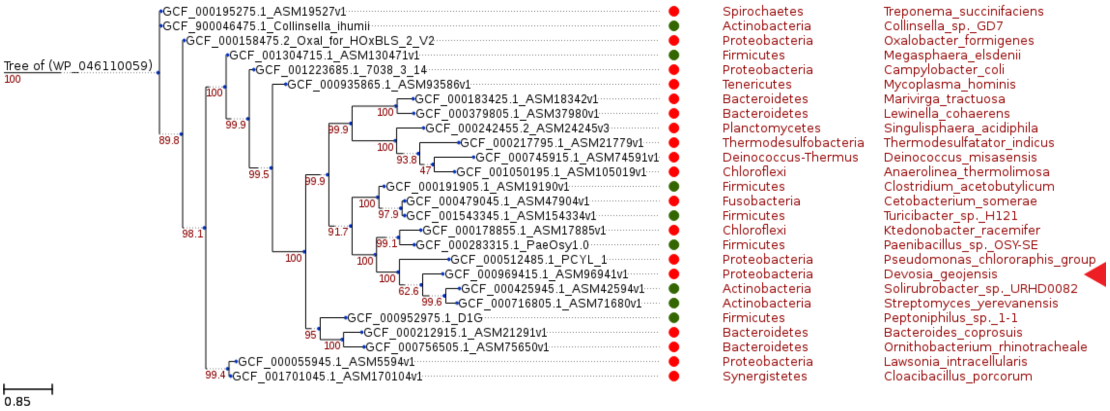
Tree of (WP_046110059)
0.85
GCF_000195275.1_ASM19527v1 Spirochaetes Treponema_succinifaciens
GCF_900046475.1_Collinsella_ihumii Actinobacteria Collinsella_sp._GD7
GCF_000158475.2_Oxal_for_HOxBLS_2_V2 Proteobacteria Oxalobacter_formigenes
GCF_001304715.1_ASM130471v1 Firmicutes Megasphaera_elsdenii
GCF_001223685.1_7038_3_14 Proteobacteria Campylobacter_coli
GCF_000935865.1_ASM93586v1 Tenericutes Mycoplasma_hominis
GCF_000183425.1_ASM18342v1 Bacteroidetes Marivirga_tractuosa
GCF_000379805.1_ASM37980v1 Bacteroidetes Lewinella_cohaerens
GCF_000242455.2_ASM24245v3 Planctomycetes Singulisphaera_acidiphila
GCF_000217795.1_ASM21779v1 Thermodesulfobacteria Thermodesulfatator_indicus
GCF_000745915.1_ASM74591v1 Deinococcus-Thermus Deinococcus_misasensis
GCF_001050195.1_ASM105019v1 Chloroflexi Anaerolinea_thermolimosa
GCF_000191905.1_ASM19190v1 Firmicutes Clostridium_acetobutylicum
GCF_000479045.1_ASM47904v1 Fusobacteria Cetobacterium_somerae
GCF_001543345.1_ASM154334v1 Firmicutes Turicibacter_sp._H121
GCF_000178855.1_ASM17885v1 Chloroflexi Ktedonobacter_racemifer
GCF_000283315.1_PaeOsy1.0 Firmicutes Paenibacillus_sp._OSY-SE
GCF_000512485.1_PCYL_1 Proteobacteria Pseudomonas_chlororaphis_group
GCF_000969415.1_ASM96941v1 Proteobacteria Devosia_geojensis
GCF_000425945.1_ASM42594v1 Actinobacteria Solirubrobacter_sp._URHD0082
GCF_000716805.1_ASM71680v1 Actinobacteria Streptomyces_yerevanensis
GCF_000952975.1_D1G Firmicutes Peptoniphilus_sp._1-1
GCF_000212915.1_ASM21291v1 Bacteroidetes Bacteroides_coprosuis
GCF_000756505.1_ASM75650v1 Bacteroidetes Ornithobacterium_rhinotracheale
GCF_000055945.1_ASM5594v1 Proteobacteria Lawsonia_intracellularis
GCF_001701045.1_ASM170104v1 Synergistetes Cloacibacillus_porcorum

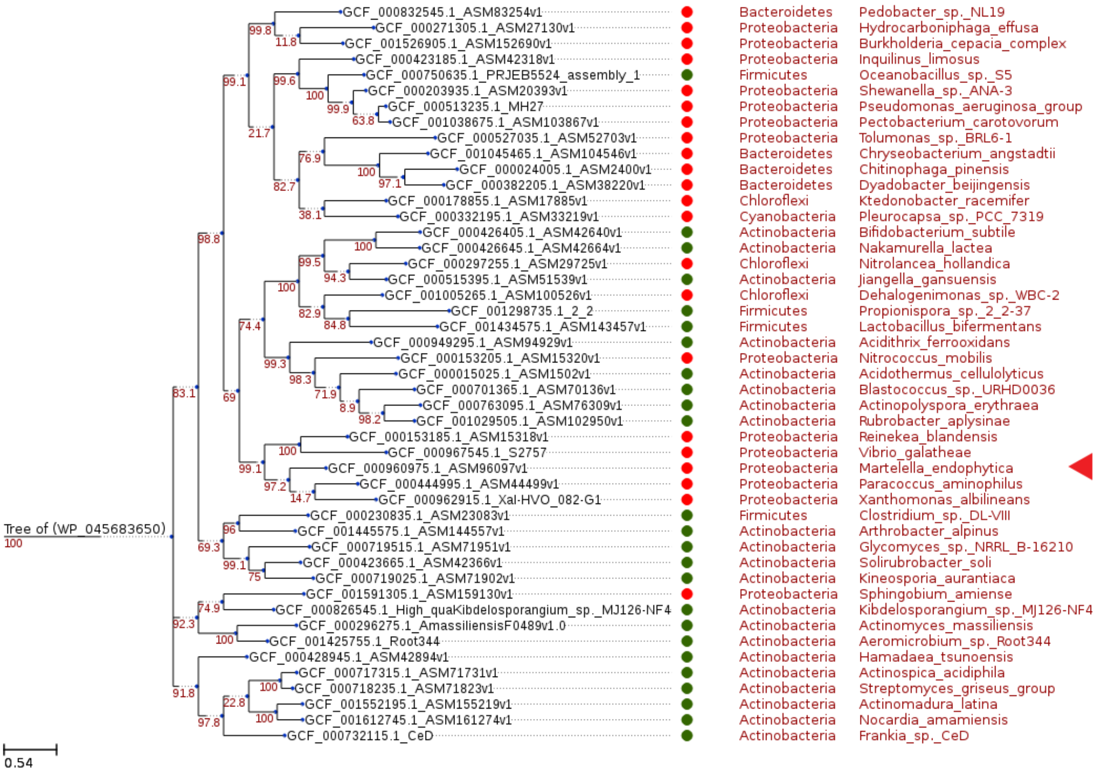
Tree of (WP_045683650)
100
GCF_000832545.1_ASM83254v1 Bacteroidetes Pedobacter_sp._NL19
GCF_000271305.1_ASM27130v1 Proteobacteria Hydrocarboniphaga_effusa
GCF_001526905.1_ASM152690v1 Proteobacteria Burkholderia_cepacia_complex
GCF_000423185.1_ASM42318v1 Proteobacteria Inquilinus_limosus
GCF_000750635.1_PRJEB5524_assembly_1 Firmicutes Oceanobacillus_sp._S5
GCF_000203935.1_ASM20393v1 Proteobacteria Shewanella_sp._ANA-3
GCF_000513235.1_MH27 Proteobacteria Pseudomonas_aeruginosa_group
GCF_001038675.1_ASM103867v1 Proteobacteria Pectobacterium_carotovorum
GCF_000527035.1_ASM52703v1 Proteobacteria Tolumonas_sp._BRL6-1
GCF_001045465.1_ASM104546v1 Bacteroidetes Chryseobacterium_angstadtii
GCF_000024005.1_ASM2400v1 Bacteroidetes Chitinophaga_pinensis
GCF_000382205.1_ASM38220v1 Bacteroidetes Dyadobacter_beijingensis
GCF_000178855.1_ASM17885v1 Chloroflexi Ktedonobacter_racemifer
GCF_000332195.1_ASM33219v1 Cyanobacteria Pleurocapsa_sp._PCC_7319
GCF_000426405.1_ASM42640v1 Actinobacteria Bifidobacterium_subtile
GCF_000426645.1_ASM42664v1 Actinobacteria Nakamurella_lactea
GCF_000297255.1_ASM29725v1 Chloroflexi Nitrolancea_hollandica
GCF_000515395.1_ASM51539v1 Actinobacteria Jiangella_gansuensis
GCF_001005265.1_ASM100526v1 Chloroflexi Dehalogenimonas_sp._WBC-2
GCF_001298735.1_2_2 Firmicutes Propionispora_sp._2_2-37
GCF_001434575.1_ASM143457v1 Firmicutes Lactobacillus_bifermentans
GCF_000949295.1_ASM94929v1 Actinobacteria Acidithrix_ferrooxidans
GCF_000153205.1_ASM15320v1 Proteobacteria Nitrococcus_mobilis
GCF_000015025.1_ASM1502v1 Actinobacteria Acidothermus_cellulolyticus
GCF_000701365.1_ASM70136v1 Actinobacteria Blastococcus_sp._URHD0036
GCF_000763095.1_ASM76309v1 Actinobacteria Actinopolyspora_erythraea
GCF_001029505.1_ASM102950v1 Actinobacteria Rubrobacter_aplysinae
GCF_000153185.1_ASM15318v1 Proteobacteria Reinekea_blandensis
GCF_000967545.1_S2757 Proteobacteria Vibrio_galatheae
GCF_000960975.1_ASM96097v1 Proteobacteria Martelella_endophytica
GCF_000444995.1_ASM44499v1 Proteobacteria Paracoccus_aminophilus
GCF_000962915.1_Xal-HVO_082-G1 Proteobacteria Xanthomonas_albilineans
GCF_000230835.1_ASM23083v1 Firmicutes Clostridium_sp._DL-VIII
GCF_001445575.1_ASM144557v1 Actinobacteria Arthrobacter_alpinus
GCF_000719515.1_ASM71951v1 Actinobacteria Glycomyces_sp._NRRL_B-16210
GCF_000423665.1_ASM42366v1 Actinobacteria Solirubrobacter_soli
GCF_000719025.1_ASM71902v1 Actinobacteria Kineosporia_aurantiaca
GCF_001591305.1_ASM159130v1 Proteobacteria Sphingobium_amiense
GCF_000826545.1_High_quaKibdelosporangium_sp._MJ126-NF4 Actinobacteria Kibdelosporangium_sp._MJ126-NF4
GCF_000296275.1_AmassiliensisF0489v1.0 Actinobacteria Actinomyces_massiliensis
GCF_001425755.1_Root344 Actinobacteria Aeromicrobium_sp._Root344
GCF_000428945.1_ASM42894v1 Actinobacteria Hamadaea_tsunoensis
GCF_000717315.1_ASM71731v1 Actinobacteria Actinospica_acidiphila
GCF_000718235.1_ASM71823v1 Actinobacteria Streptomyces_griseus_group
GCF_001552195.1_ASM155219v1 Actinobacteria Actinomadura_latina
GCF_001612745.1_ASM161274v1 Actinobacteria Nocardia_amamiensis
GCF_000732115.1_CeD Actinobacteria Frankia_sp._CeD
0.54

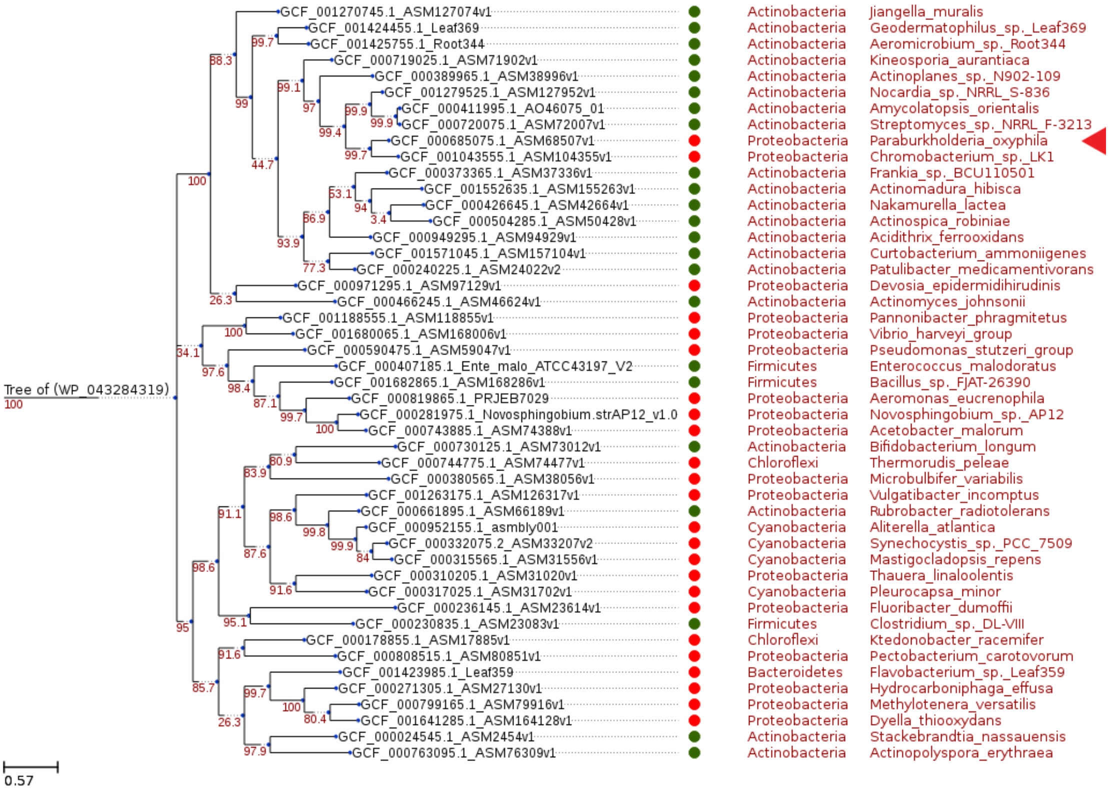
Tree of (WP_043284319)
100
GCF_001270745.1_ASM127074v1 Actinobacteria Jiangella_muralis
GCF_001424455.1_Leaf369 Actinobacteria Geodermatophilus_sp._Leaf369
GCF_001425755.1_Root344 Actinobacteria Aeromicrobium_sp._Root344
GCF_000719025.1_ASM71902v1 Actinobacteria Kineosporia_aurantiaca
GCF_000389965.1_ASM38996v1 Actinobacteria Actinoplanes_sp._N902-109
GCF_001279525.1_ASM127952v1 Actinobacteria Nocardia_sp._NRRL_S-836
GCF_000411995.1_AO46075_01 Actinobacteria Amycolatopsis_orientalis
GCF_000720075.1_ASM72007v1 Actinobacteria Streptomyces_sp._NRRL_F-3213
GCF_000685075.1_ASM68507v1 Proteobacteria Paraburkholderia_oxyphila
GCF_001043555.1_ASM104355v1 Proteobacteria Chromobacterium_sp._LK1
GCF_000373365.1_ASM37336v1 Actinobacteria Frankia_sp._BCU110501
GCF_001552635.1_ASM155263v1 Actinobacteria Actinomadura_hibisca
GCF_000426645.1_ASM42664v1 Actinobacteria Nakamurella_lactea
GCF_000504285.1_ASM50428v1 Actinobacteria Actinospica_robiniae
GCF_000949295.1_ASM94929v1 Actinobacteria Acidithrix_ferrooxidans
GCF_001571045.1_ASM157104v1 Actinobacteria Curtobacterium_ammoniigenes
GCF_000240225.1_ASM24022v2 Actinobacteria Patulibacter_medicamentivorans
GCF_000971295.1_ASM97129v1 Proteobacteria Devosia_epidermidihirudinis
GCF_000466245.1_ASM46624v1 Actinobacteria Actinomyces_johnsonii
GCF_001188555.1_ASM118855v1 Proteobacteria Pannonibacter_phragmitetus
GCF_001680065.1_ASM168006v1 Proteobacteria Vibrio_harveyi_group
GCF_000590475.1_ASM59047v1 Proteobacteria Pseudomonas_stutzeri_group
GCF_000407185.1_Ente_malo_ATCC43197_V2 Firmicutes Enterococcus_malodoratus
GCF_001682865.1_ASM168286v1 Firmicutes Bacillus_sp._FJAT-26390
GCF_000819865.1_PRJEB7029 Proteobacteria Aeromonas_eucrenophila
GCF_000281975.1_Novosphingobium.strAP12_v1.0 Proteobacteria Novosphingobium_sp._AP12
GCF_000743885.1_ASM74388v1 Proteobacteria Acetobacter_malorum
GCF_000730125.1_ASM73012v1 Actinobacteria Bifidobacterium_longum
GCF_000744775.1_ASM74477v1 Chloroflexi Thermorudis_peleae
GCF_000380565.1_ASM38056v1 Proteobacteria Microbulbifer_variabilis
GCF_001263175.1_ASM126317v1 Proteobacteria Vulgatibacter_incomptus
GCF_000661895.1_ASM66189v1 Actinobacteria Rubrobacter_radiotolerans
GCF_000952155.1_asmbly001 Cyanobacteria Aliterella_atlantica
GCF_000332075.2_ASM33207v2 Cyanobacteria Synechocystis_sp._PCC_7509
GCF_000315565.1_ASM31556v1 Cyanobacteria Mastigocladopsis_repens
GCF_000310205.1_ASM31020v1 Proteobacteria Thauera_linaloolentis
GCF_000317025.1_ASM31702v1 Cyanobacteria Pleurocapsa_minor
GCF_000236145.1_ASM23614v1 Proteobacteria Fluoribacter_dumoffii
GCF_000230835.1_ASM23083v1 Firmicutes Clostridium_sp._DL-VIII
GCF_000178855.1_ASM17885v1 Chloroflexi Ktedonobacter_racemifer
GCF_000808515.1_ASM80851v1 Proteobacteria Pectobacterium_carotovorum
GCF_001423985.1_Leaf359 Bacteroidetes Flavobacterium_sp._Leaf359
GCF_000271305.1_ASM27130v1 Proteobacteria Hydrocarboniphaga_effusa
GCF_000799165.1_ASM79916v1 Proteobacteria Methylotenera_versatilis
GCF_001641285.1_ASM164128v1 Proteobacteria Dyella_thiooxydans
GCF_000024545.1_ASM2454v1 Actinobacteria Stackebrandtia_nassauensis
GCF_000763095.1_ASM76309v1 Actinobacteria Actinopolyspora_erythraea
0.57

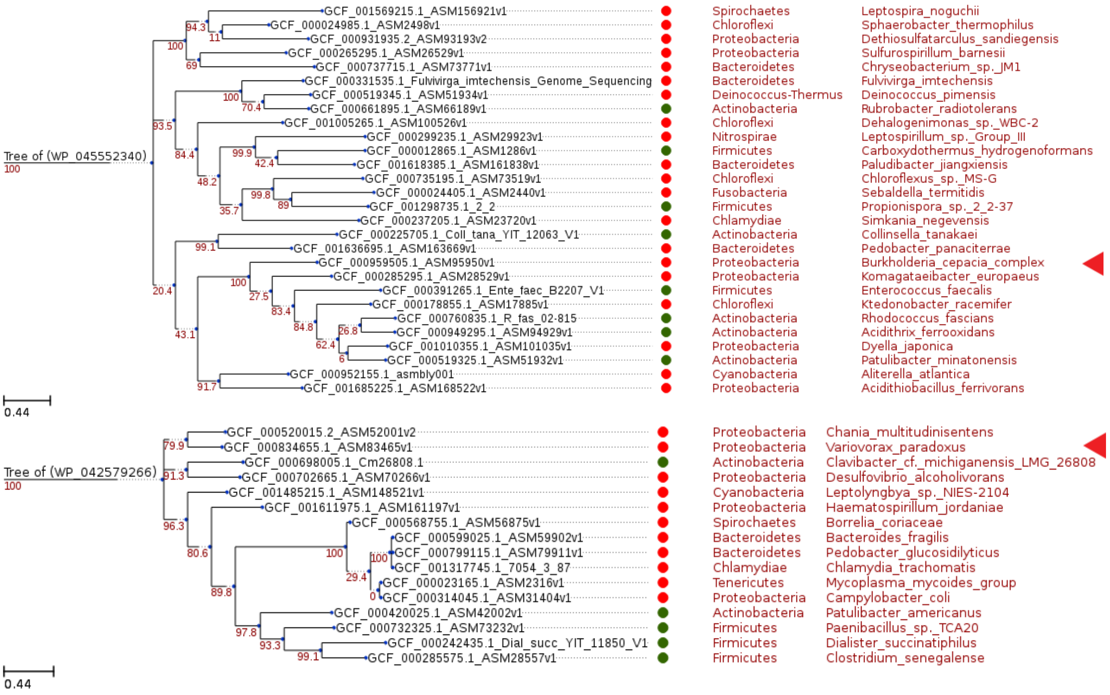

Supplementary Figure 1 | Protein phylogenetic trees. Phylogenetic trees were built for the proteobacterial proteins that show the highest sequence identity to *Streptomyces* ARG proteins, the proteins encoded in neighboring area of resistance gene *pac*, and proteins involved in *cmx* "carry back". The position of the query sequence was marked by red triangle in the trees. Gram-positive and Gram-negative bacteria are marked in red and blue respectively.

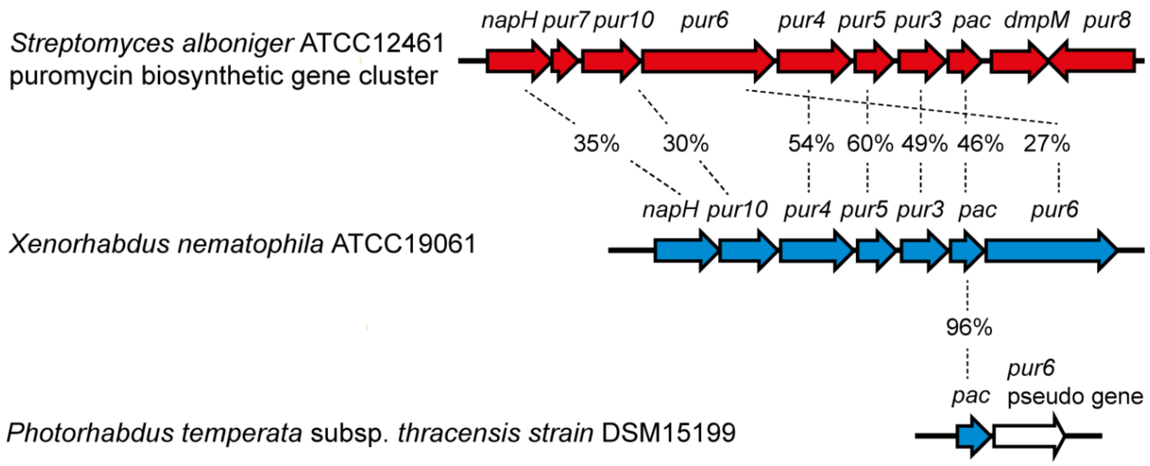

Supplementary Figure 2 | Puromycin resistance gene *pac* and neighbouring genes in actinobacteria and proteobacteria. The puromycin biosynthetic gene cluster in puromycin producer is composed of ten genes (red). Homologues (protein sequence identity value indicated) of seven of them including the resistance gene *pac* (WP_046974149.1) were found in proteobacteria (blue). A standalone *pac* was also found in proteobacteria (blue). The products of all these genes were more similar to actinobacterial proteins than to proteins from any other phyla, suggesting they were co-transferred from actinobacteria to proteobacteria (Supplementary Fig. 1).

```
-----------MPFALYMLALAVFVMGTSEFMLAGLLPAIATELDVSVGTAGLLTSAFAVGMVVGAPVMAAFARRWPPRLTLIVCLLVFAGSHVIGAMTP
-----------MPFAIYVLGLAVFAQGTSEFMLSGLVEDMAADFGVSLQAVGSLTSAFAVGMIIGAPLVAMLSLRWPRRRALLAFLITFLLAHVVGALTN
-----------MPLPLYLLAVAVCAMGTSEFMLAGLVPDIASDLGVTVGTAGTLTSAFATGMIVGAPLVAALARTWPRRSSLLGFILAFAAAHAVGAGTT
MSSKNFSWRYSLAATVLLLSPFDLLASLGMDMYLPAVPFMPNALGTTASTIQLTLTTYLVMIGAGQLLFGPLSDRLGRRPVLLGGLAYVVASMGLALTS

VFSLLLITRVLSALANAGFLAVALSTATTLVPANQKG-RALSILLSGTTIATVVGVPAGALLGTALGWRTTFWAIAILCIPAAVGVIRGVTNNVGRSETS
SFEVLFVTRVIAALANAGFLAVGLATAASMAGPQAKG-RATSILLSGVTLACIAGVPLGAVLSEVWGWRSAFWGVAIISAPAVFAVLWSVP-----ATPV
SFPVLVACRVVAALANAGFLAVALTTAAALVPADKQG-RALAVLLSGTTVATVAGVPGGSLLGTWLGWRATFWAVAVCCLPAAFGVLKAIP--AGRATAA
SAEVFLGLRILQACGASACLVSTFATVRDIYAGREESNVIYGILGSMLAMVPAVGPLLGALVDMWLGWRAIFAFLGLGMIAASAAAWRFWP----ETRVQ

ATS-PRLRVELSQLATPRLILAMALGALINGGTFAAFTFLAPIVTETAGLAEAWVSVALVMFGIGSFLGVTIAGRLSDQRPG-LVLAVGGPLLLTGWIVL
DPSAPSVRHELRALRTPKVGVTLLLGALVNGATFCSFTYLAPVITNVTELSSGWVPATLALFGIGSFVGVNVGGRLADTRPG-QVLAVGGAALLAGWLVF
ATGGPPLRVELAALKTPRLLLAMLLGALVNAATFASFTFLAPVVTDTAGLGDLWISVALVLFGAGSFAGVTVAGRLSDRRPA-QVLAVAGPLLLVGWPAL
RVAGLQWSQLLLPVKCLNFWLYTLCYAAGMGSFFVFFSIAPGLMMGRQGVSQLGFSLLFATVAIAMVFTARFMGRVIPKWGSPSVLRMGMGCLIAGAVLL

AVVASHPVALIV-LVLVQGFLSFGVGSTLITRVLYAASGAPTMGGSYATAALNIGAAAGPVLGALGLATGLGLLAPVWVASVLTAIALVIMLLTRRALTK
ALTAGNPVVALV-LVFVQGALSFGVGSTLIAQALYTASDAPTLAGGFATAALNVGAAAGPALGGLALGAEFGYRSPLVVSAVLVATALVLAGVARAAGVT
AMLADRPVALLT-LVFVQGALSFALGSTLITRVLYEAAGAPTMAGSYATAALNVGAAAGPLVAATTLGHTTGNLGPLWASGLLVAVALLVAFPFRTVITT
AITEIWALQSVLGFIAPMWLVGIGVATAVSVAPNGALRGFDHVAGTVTAVYFCLGGVLLGSIGTLIISLLPRNTAWPVVVYCLTLATVVLGLSCVSRVKG

TAAEAN-----------------       multi-phylum Cmx (WP_005297378)
GAKADAAATENPPTGRPVEARS-       S. venezuelae self-protecting chloramphenicol efflux pump (WP_015032122)
AAPADATR---------------       S. lividans confirmed chloramphenicol efflux pump (P31141)
SRGQGEHDVVALQSAESTSNPNR       Pseudomonas aeruginosa CmlA (WP_000095725)
```

Supplementary Figure 3 | Alignment of the multi-phylum Cmx, and three other confirmed chloramphenicol resistance pumps. Alignment was performed using software Vector NTI. Non-similar residues are shown as black letters on a white background; weakly similar residues are shown as green letters on a white background; conservative residues are shown as black letters on a blue background; block of similar residues are shown as black letters on a green background; identical residues are shown as red letters on a yellow background. The multi-phylum Cmx showed 63% identity (100% coverage) to the chloramphenicol efflux pump (P31141) from the model actinobacterium *Streptomyces lividans* 66, and 52% identity (99% coverage) to the self-protecting efflux pump (WP_015032122.1) from the chloramphenicol producer *Streptomyces venezuelae*. Meanwhile, it showed only 29% identity (27% coverage) to CmlA (WP_000095725), a confirmed proteobacterial chloramphenicol efflux pump.

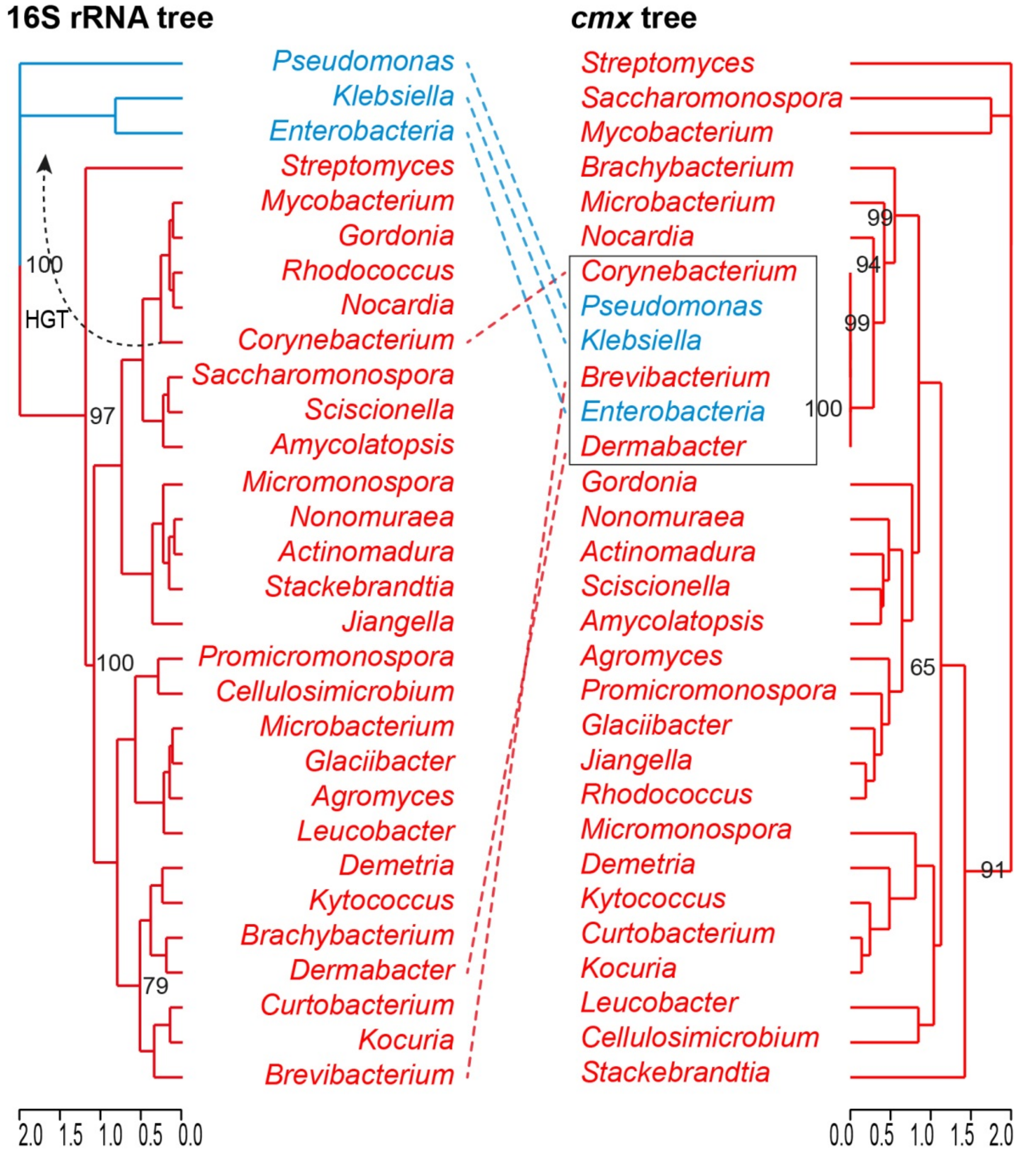

Supplementary Figure 4 | A full tanglegram of the phylogeny of the genus representative hits of *cmx* and the host phylogeny of their corresponding 16S rRNA sequences. The red labels show the Gram-positive actinobacteria while the blue labels illustrate the Gram-negative proteobacteria. *cmx* is said to have a hit in a genome if the genome has a Cmx protein (WP_005297378.1) sequence match with e-value of 10E-50 or less, 30% BLAST identity and 80% coverage. The best hit per genus is taken as the representative hit. The hits with over 99% identity to WP_005297378.1 are highlighted with a frame.

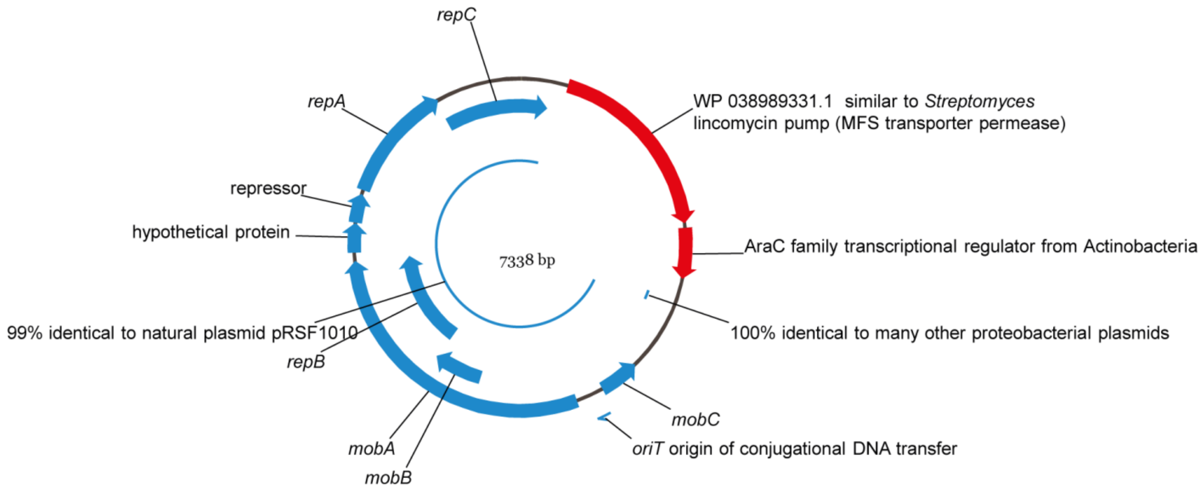

Supplementary Figure 5 |WP_038989331.1 gene from *Salmonella enterica* PS01. The predicted transporter protein WP_038989331.1 is 50% identical to the self-protecting lincomycin pump, LmrA (CAA42550), from *Streptomyces lincolnensis*. Its gene is found on a plasmid from *Salmonella enterica* PS01. Similar plasmids were also found from *Salmonella enterica* subsp. enterica serovar typhimurium str. DT104 (NZ_CTFZ01000046.1:1-9225) and *Escherichia coli* EC2_1 (NZ_JWKJ01000106.1:1-7466). The WP_038989331.1 gene and the adjacent regulator gene showed actinobacterial sequence signature (highlighted in red), suggesting that they were transferred from actinobacteria recently. The region from *mobC* to *repC* is 99% identical to the broad-host-range proteobacterial plasmid pRSF1010 (highlighted in blue). These elements are responsible for the ability of pRSF1010 to be mobilized by conjugation from *E.coli* into *Streptomyces* and *Mycobacterium* (helped by conjugative plasmid) and stably maintained there.

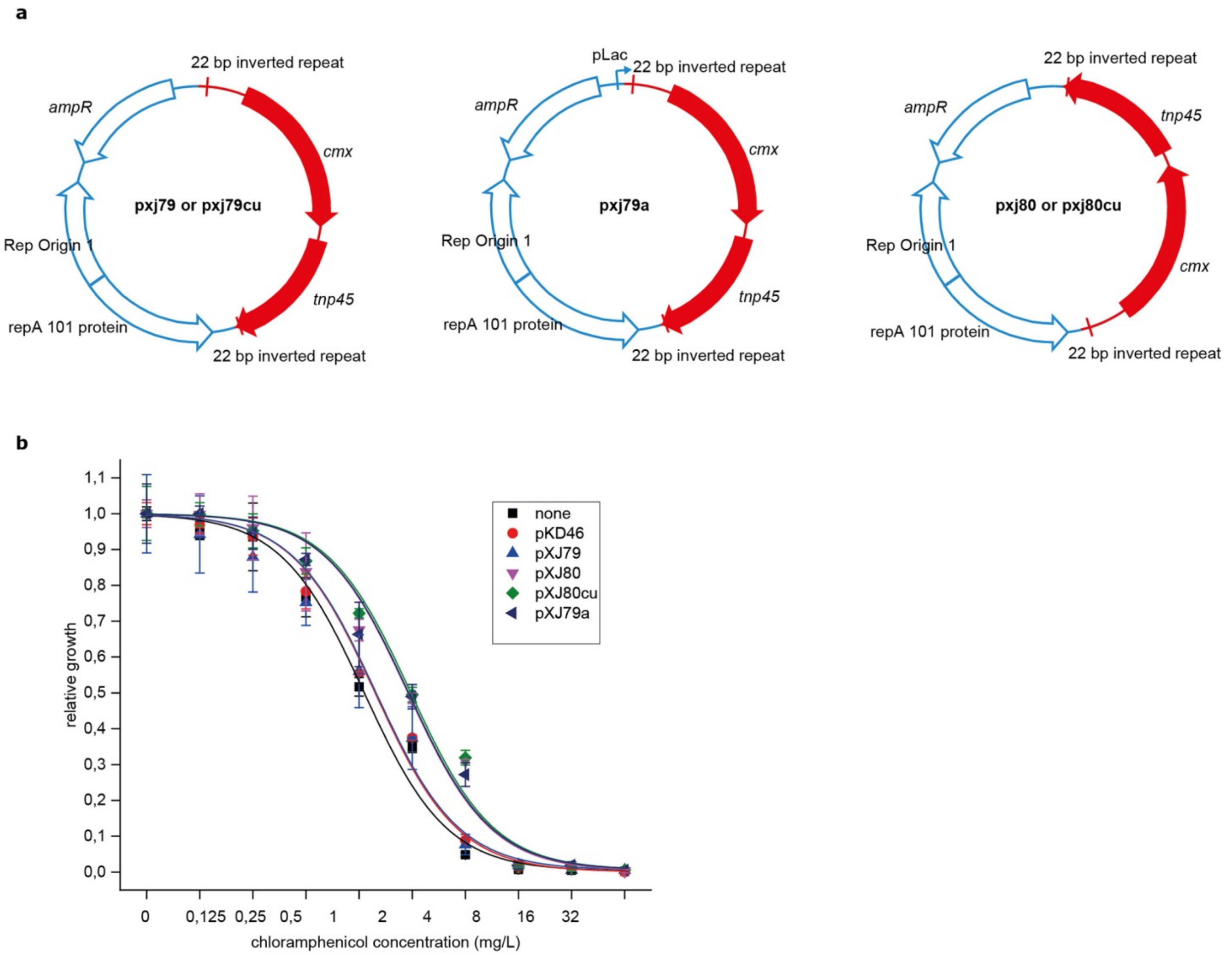

Supplementary Figure 6 | Cloning of *cmx* transposon and its chloramphenicol resistance activity in *E.coli*. a, Cloning of *cmx* transposon. The *cmx* transposon (highlighted in red) was PCR amplified from *P. aeruginosa* PA7 by primer xj143 (as both forward and reverse primer). Plasmid backbone (marked in blue) was amplified from plasmid pKD46 by primer xj144 and xj145. The two PCR products were assembled by Gibson reaction, transferred into *E. coli* DH5α and selected on Amp plate. Insertion direction was determined by sequencing with primer xj146. The resulted plasmids were named as pXJ79 and pXJ80. Plasmids pXJ79cu and pXJ80cu were constructed likewise and the *cmx* transposon was amplified from *C. urealyticum* DSM 7109. Plasmid pXJ79 was amplified with primer xj177and xj178, and the product was recirculated by Gibson reaction, generating pXJ79a. By this way promoter pLac (without lacO) was inserted in front of the transposon. **b,** Dose-response curves of chloramphenicol on *E.coli* DH5α with cloned *cmx* transposon. Concentrations of chloramphenicol

were included at twofold serial dilutions. Growth was conducted in quadruplicate 50 µl volumes for each antibacterial concentration in 96-well plates. After 48 h of incubation at 30°C, the OD600 was determined using a plate reader. Quadruplicate values for each drug condition were averaged and then normalized to growth levels without added drug (error bar: s.d.; n=4). Increased tolerance towards chloramphenicol was observed when a promoter was provided in front of the transposon (pXJ79a). A similar level of resistance was also observed when the transposon was inserted in the same direction as upstream native genes (pXJ80), suggesting that *cmx* can be functionally expressed in the new host by read-through of transcription from upstream native genes.

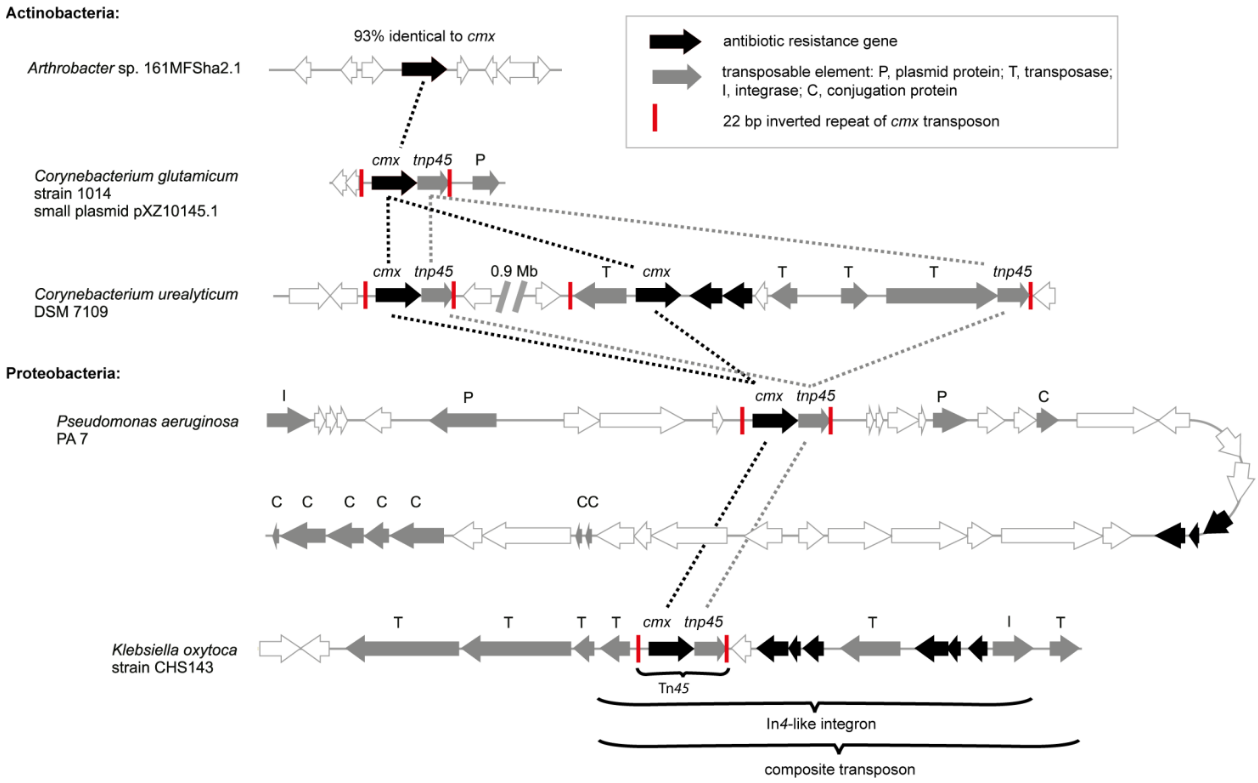

Supplementary Figure 7 | Examples of *cmx* transposon and its context in different hosts. Antibiotic resistance and mobile element genes are colored as black and grey respectively. Mobile element types are indicated. A gene 93% identical to *cmx* is found in *Arthrobacter* sp. 161MFSha2.1. No *tnp45* gene is found in the same genome. On *C. glutamicum* plasmid pXZ10145.1, a basic form of *cmx* transposon with only *cmx* and *tnp45* inside was found. On *C. urealyticum* DSM 7109 chromosome, the *cmx* transposon duplicated, and the second copy has a new transposon Tn*5393* carrying two aminoglycoside phosphotransferases inserted between *cmx* and *tnp45*. On *P. aeruginosa* PA7 chromosome, the *cmx* transposon was found within a 53 kb integrated plasmid with conjugation proteins, transposases, integrase and four other ARGs. In *K. oxytoca*, the *cmx* transposon is located in an In*4*-like integron and a composite transposon.

Supplementary Table 1 | primer sequences used in cloning and sequencing

Primer name	Primer sequence 5’-3’
**Xj143**	TAGGGATAACAGGGTAATCTAGCTGTGATGTCCAGGGACGTTGTT
**Xj144**	ATTACCCTGTTATCCCTAAAATTGGAATCAGGTTTGTGCC
**Xj145**	ATTACCCTGTTATCCCTACGAGTTCCGTGCCGGTTG
**Xj146**	AATACCGCGCCACATAGCAG
**Xj171.1**	CAACAGAGCCTGGCGTAATAGCGAAGAGGC
**Xj172**	CACCGTCACCAGCTGTTTCCTGTGTGAAATTGTTA
**Xj173**	GGAAACAGCTGGTGACGGTGTTCGGCATTC
**Xj174.1**	TATTACGCCAGGCTCTGTTGCAAAGATTGGC
**Xj177**	TTATGCTTCCGGCTCGTATGTTGTGTGGATTCACTTTTTCTTCACAACCGG
**Xj178**	ACATACGAGCCGGAAGCATAAAGTGTAAAGTCATAATAAATCGATGCAGGTGG
**Xj130**	GGAAACAGCTATGCCTTTTGCCCTCTACATGC
**Xj131**	TGCGTAAGGAGGATAGCCGTCTTCGACAATCAG
**Orf5-f-1**	TGCCACCCGAACCTGCG
**Tnp6100-r-1**	ATCGGATAGCGACAATACCAG
